# A novel human pain insensitivity disorder caused by a point mutation in *ZFHX2*

**DOI:** 10.1093/brain/awx326

**Published:** 2017-12-14

**Authors:** Abdella M Habib, Ayako Matsuyama, Andrei L Okorokov, Sonia Santana-Varela, Jose T Bras, Anna Maria Aloisi, Edward C Emery, Yury D Bogdanov, Maryne Follenfant, Sam J Gossage, Mathilde Gras, Jack Humphrey, Anna Kolesnikov, Kim Le Cann, Shengnan Li, Michael S Minett, Vanessa Pereira, Clara Ponsolles, Shafaq Sikandar, Jesus M Torres, Kenji Yamaoka, Jing Zhao, Yuriko Komine, Tetsuo Yamamori, Nikolas Maniatis, Konstantin I Panov, Henry Houlden, Juan D Ramirez, David L H Bennett, Letizia Marsili, Valeria Bachiocco, John N Wood, James J Cox

**Affiliations:** 1 Molecular Nociception Group, Wolfson Institute for Biomedical Research, University College London, London, UK; 2 College of Medicine, Member of Qatar Health Cluster, Qatar University, Doha, Qatar; 3 Department of Molecular Neuroscience, Institute of Neurology, University College London, London, UK; 4 Department of Medicine, Surgery and Neuroscience, University of Siena, via Aldo Moro, 2, Siena, Italy; 5 Department of Biochemistry, Molecular Biology and Immunology, Faculty of Medicine, University of Granada, Granada, Spain; 6 National Institute for Basic Biology, Okazaki, Japan; 7 Department of Genetics, Evolution and Environment, University College London, London, UK; 8 Medical Biology Centre, School of Biological Sciences, Queen’s University Belfast, Belfast, UK; 9 Nuffield Department of Clinical Neurosciences, University of Oxford, Oxford, UK; 10 Department of Physical Sciences, Earth and Environment, University of Siena, Siena, Italy

**Keywords:** pain insensitivity, Mendelian, dorsal root ganglia, transcription factor

## Abstract

Chronic pain is a major global public health issue causing a severe impact on both the quality of life for sufferers and the wider economy. Despite the significant clinical burden, little progress has been made in terms of therapeutic development. A unique approach to identifying new human-validated analgesic drug targets is to study rare families with inherited pain insensitivity. Here we have analysed an otherwise normal family where six affected individuals display a pain insensitive phenotype that is characterized by hyposensitivity to noxious heat and painless bone fractures. This autosomal dominant disorder is found in three generations and is not associated with a peripheral neuropathy. A novel point mutation in *ZFHX2*, encoding a putative transcription factor expressed in small diameter sensory neurons, was identified by whole exome sequencing that segregates with the pain insensitivity. The mutation is predicted to change an evolutionarily highly conserved arginine residue 1913 to a lysine within a homeodomain. Bacterial artificial chromosome (BAC) transgenic mice bearing the orthologous murine p.R1907K mutation, as well as *Zfhx2* null mutant mice, have significant deficits in pain sensitivity. Gene expression analyses in dorsal root ganglia from mutant and wild-type mice show altered expression of genes implicated in peripheral pain mechanisms. The ZFHX2 variant and downstream regulated genes associated with a human pain-insensitive phenotype are therefore potential novel targets for the development of new analgesic drugs.

## Teaser

Studies of monogenic heritable pain disorders provide valuable insights into human pain mechanisms. Habib *et al.* show that a point mutation in the gene *ZFHX2* causes an autosomal dominant form of pain insensitivity. Modulating ZFHX2 and/or downstream genes may present a new strategy for the treatment of chronic pain.

## Introduction

Congenital hypoalgesia, in which patients are born with a reduced capacity to detect tissue-damage causing stimuli, is a rare inherited phenotype. In the most extreme forms, patients can suffer from a complete inability to perceive noxious stimuli which leads to dangerous, but painless, injuries such as biting off of fingertips, lips and the tongue, and frequent bone fractures (Nahorski *et al.*, 2015). Recessive loss of function mutations in the voltage-gated sodium channel gene *SCN9A*, which encodes Na_v_1.7, are a major cause of this form of pain insensitivity (OMIM 243000) (Cox *et al.*, 2006; Habib *et al.*, 2015). A second, less frequent cause, are dominant gain of function mutations in the Na_v_1.9 channel, which to date have been reported in only four families worldwide (OMIM 615548) (Leipold *et al.*, 2013; Woods *et al.*, 2015; Phatarakijnirund *et al.*, 2016). In both of these channelopathy disorders there is an absence of an associated peripheral neuropathy with the phenotype related to dysfunction of voltage-gated sodium channels within the intact sensory neurons. In the case of Na_v_1.7, this dysfunction has been shown to result in an upregulation of endogenous opioids, with the painlessness capable of being partially reversed in both humans and mice through a systemic infusion of naloxone, an opioid receptor antagonist (Minett *et al.*, 2015).

Inherited pain insensitivity can also arise through congenital absence or a progressive degeneration of sensory and autonomic neurons, the so-called hereditary sensory and autonomic neuropathies (HSANs) (Rotthier *et al.*, 2012). This heterogeneous group of disorders differ in their age of onset (juvenile or adult) and degree of motor, sensory and autonomic disturbances, which correlates largely with the subtypes of neurons that are affected. In the case of HSAN IV (OMIM 256800) and HSAN V (OMIM 608654), in which there are recessive loss of function mutations in the genes encoding the nerve growth factor (NGF) receptor (TRK-A) and NGF, respectively, there is a congenital absence of damage- and temperature sensing neurons and a deficient innervation of sweat glands that can lead to anhidrosis (Indo *et al.*, 1996; Einarsdottir *et al.*, 2004; Carvalho *et al.*, 2011). The functions of HSAN genes are diverse, ranging from neurotrophic functions (*NTRK1*/TRK-A and *NGF*), sphingolipid metabolism (*SPTLC1* and *SPTLC2*), structural integrity of the endoplasmic reticulum (*ATL1*) and Golgi apparatus (*RETREG1*/FAM134B) and vesicular trafficking (*RAB7A* and *KIF1A*) (Rotthier *et al.*, 2012). Recently, several HSAN mutations were also reported in *PRDM12*, an epigenetic regulator that is expressed in nociceptors and their progenitors (OMIM 616488). Loss of function of PRDM12 leads to a severe congenital peripheral neuropathy, with a lack of nerve fibres crossing the dermal-epidermal border as assessed by staining of a skin biopsy, and a consequent pain-insensitive phenotype (Chen *et al.*, 2015).

Here, we have studied a family where congenital hypoalgesia is present in six affected individuals across three generations. Through a combination of whole exome sequencing and mouse model analyses we have shown that the genetic defect resides in *ZFHX2* (Zinc finger homeobox 2), a nociceptor-expressed transcriptional regulator. Gene expression analyses show that the missense mutation in *ZFHX2* causes significant gene deregulation within dorsal root ganglia (DRG), helping to explain the observed pain insensitive phenotype.

## Materials and methods

### Study subjects

The family was identified in Italy and was previously reported (Spinsanti *et al.*, 2008), although the full extent of the phenotype was only elucidated following publication. The family consists of a mother (aged 78), her two daughters (aged 52 and 50) and their children (two boys and one girl, aged 24, 21 and 16, respectively). In different severity and forms, these subjects presented a low ability to sense pain, to experience temperature and to sweat. Indeed, the members suffered unnoticed bone fractures in, for example, the arms and legs (Supplementary Table 1) and cutaneous injuries, but occasionally suffered from headaches and visceral pains. In addition, members manifested a low sensitivity to capsaicin (chilli pepper) and a normal or high sensitivity to odours. Sometimes the intense sensations were accompanied by autonomic reactions such as vomiting and fainting. Episodes of unexplained hyperthermia occasionally occurred. Cognitive ability and motor performances were normal or high. Written informed consent was obtained from all participants and the study was approved by the ethics committee at University College London, UK, with sensory phenotype assessments at the University of Siena, Italy.

### Sensory phenotype assessment methods

#### Tender points

The number of positive tender points was assessed with a pressure algometer (footplate surface, 1 cm^2^; scale range, 0–10 kg); the point was considered positive if the subject reported pain with a pressure lower than 4 kg. The pressure was applied once to 18 tender points (9 + 9) and 10 control points (5 + 5) (Wolfe *et al.*, 1990). The pressure gauge was advanced at a rate of ∼1 kg/s and stopped when the subject declared that the pressure pain level had been reached.

#### Thermal detection pain threshold

Superficial cold and heat pain thresholds were determined with a thermal stimulator (Medical Instruments Facilities). Four cold and four heat stimuli were delivered on the thenar eminence of the dominant hand at 1.5°C/s starting from 32°C and passing to −10°C or 50°C, respectively, and the thresholds were expressed as the mean values of the four responses. The subject was asked to press a button to interrupt the stimulus when the thermal sensation became a nociceptive sensation.

#### Cold pressor test

Tonic cold pain was elicited by the cold pressor test (Wolf and Hardy, 1941) and pain onset and pain tolerance latencies (in seconds) were determined. Subjects kept their non-dominant hand fully immersed in a plastic box containing water at 37°C for 1 min, then immersed the same hand in another box containing cold water (0–1°C) and ice grains, and kept it there as long as possible (pain tolerance latency). During hand immersion, the subjects reported when the sensation became painful (pain threshold latency).

#### Mechanical threshold detection

A standard set of von Frey filaments, round tip ∼0.5 mm (evaluator size 1.65–6.65; target force 0.008–300 g, Touch Test Sensory Evaluators), were administered in ascending and descending order five times (randomly) on the volar surface of the non-dominant forearm in an area along the midline, approximately midway from the wrist crease to elbow crease. The subject was asked to communicate when a touch sensation was perceived.

#### Mechanical pain threshold detection

A series of modified von Frey filaments with a sharp tip (0.25 mm, target force 4.56–6.45 i.e. 4–180 g) able to activate the cutaneous nociceptors (Rolke *et al.*, 2006), were applied till bending, in ascending and descending order five times (randomly) on the volar part of the arm with a contact of 1 s. The subject was asked to quantify the intensity of the painful/pleasurable sensation (VAS 0-100).

#### Capsaicin test

The volar surface of the non-dominant forearm in an area along the midline, approximately midway from the wrist to the elbow crease, an area of ∼4 cm^2^, was chosen for testing. From the centre of this area, radial spokes were traced out towards the periphery for ∼6 cm. The proband was invited to fix her eyes on a precise point of the wall during each test when requested.

Basal responsiveness in the area was measured first. For touch evoked sensation, cotton wool was gently swept at a rate of 1 cm/s along each radial spoke starting at the point nearest to the elbow and moving to the centre point. The proband was asked to report if the stimulus was nothing, tactile, unpleasant, painful or other. For mechanical pain detection, the von Frey filament (6.45) was applied along each radial spoke 1 cm apart (5 min after the cotton wool test).

Ten minutes later, a solution of capsaicin (0.250 µg capsaicin in 250 µl saline) was injected intradermally (25 µl) into the centre point of the map using a 0.5 ml insulin syringe using a 28-gauge needle.

The subject was asked to report the onset of whichever sensation and if painful to rate its intensity (VAS 0-100) and its unpleasantness (VAS 0-100) and all changes across time in order to build up the temporal profile. Cotton wool and von Frey filaments were used as described above following capsaicin injection.

### Skin biopsy

A punch skin biopsy was taken from 10 cm above the lateral malleolus of the leg of the proband and fixed overnight with 2% periodate-lysine-paraformaldehyde and preserved in sucrose before blocked and processed into 50 µM sections. Nerve fibres were stained using rabbit anti-PGP (protein gene product) 9.5 antibody (1:2000; Ultraclone Ltd) and Cy3 anti-rabbit (1:500; Jackson Immunoresearch). By means of a Zeiss LSM 710 confocal microscope, *z*-stacks (2-μm intervals), maximum intensity projections were generated with a Plan-Apochromat objective at ×20 magnification (Carl Zeiss MicroImaging GmbH). Analysis was performed as per published guidelines (Lauria *et al.*, 2010). PGP9.5-positive nerve fibres crossing the dermal-epidermal junction were counted and intraepidermal nerve fibre density counts are given in number of fibres per millimetre of skin.

### Whole exome sequencing

For enrichment of exons and flanking intronic sequences we used the Illumina TruSeq DNA Sample Prep Kit and Exome Enrichment Kit (Individuals III-1 and III-2) and the Agilent Human SureSelect V4 kit (Individuals I-2, II-2, II-4 and III-3). We performed 100 bp paired-end runs on a Genome Analyzer HiSeq 2000 system (Illumina) generating sequences of 2 (Individual III-1), 2.3 (Individual III-2), 6.0 (Individual I-2), 7.4 (Individual II-2), 6.9 (Individual II-4) and 7.0 (Individual III-3) Gb. This amount of data resulted in the following percentages of targets being covered at ≥10×: 83.3 (Individual III-1), 83.1 (Individual III-2), 93.4 (Individual I-2), 98.3 (Individual II-2), 98.1 (Individual II-4) and 98.5 (Individual III-3). Sequence alignment and variant calling was performed against the reference human genome assembly (hg19) by using the Burrows-Wheeler Aligner (Li and Durbin, 2009) and the Genome Analysis Toolkit (McKenna *et al.*, 2010; DePristo *et al.*, 2011). Format conversion and indexing were performed with the Picard software. Single nucleotide variants and small insertions and deletions were checked against established databases (1000 Genomes Project and dbSNP v.142). Variants were further checked using the ExAC browser, dbSNP v.147 and in our in-house database of sequencing data for other diseases (*n* > 2000). The protein coding effects of variants was predicted using SIFT, PolyPhen-2 and M-CAP. Splicing changes were analysed using the NNSPLICE Splice Site Predictor. Novel variants were verified by Sanger sequencing and checked to see if they segregated in all six affected individuals (primers available on request).

### Animal behaviour tests

All experiments were performed in accordance with the UK Animals (Scientific Procedures) Act 1986 with prior approval under a Home Office project licence (PPL 70/7382). Mice were kept on a 12-h light/dark cycle and provided with food and water *ad libitum*. Global *Zfhx2* knockout mice were previously generated (Komine *et al.*, 2012) and imported to the UK from the RIKEN Bio Resource Center (BRC No. 02262: B6.129S-Zfhx2<tm3Ymri>). Zfhx2 p.R1907K BAC (bacterial artificial chromosome) transgenic mice were generated by Cyagen Biosciences Inc (further details in the Supplementary material). Experiments were conducted using both male and female wild-type littermate and knockout/transgenic mice, all of which were at least 7 weeks old when tested. Observers who performed behavioural experiments were blind to the genotype of the animals. Mechanical nociceptive thresholds were measured using a modified version of the Randall Selitto test that applies pressure to the tail via a 3 mm^2^ blunt conical probe with a 500-g cut-off (Randall and Selitto, 1957; Minett *et al.*, 2011). Touch perception was measured using the up-down method for obtaining the 50% threshold using von Frey hairs, as previously described (Chaplan *et al.*, 1994; Minett *et al.*, 2011). Thermal nociceptive thresholds were determined by measuring paw-withdrawal latency using the Hargreaves’ apparatus (Hargreaves *et al.*, 1988; Minett *et al.*, 2011) with a ramp of 1.5°C/s and a 30-s cut-off and also by use of the 50°C hot-plate test (Eddy and Leimbach, 1953). The response to mild cooling was measured using the acetone evaporation test (Minett *et al.*, 2011) and the response to noxious cold measured using the cold plantar assay (Brenner *et al.*, 2012). The rotarod test was performed as described in Stirling *et al.* (2005).

### *In vivo* electrophysiology

Electrophysiological recordings were performed by an experimenter blind to genotype. Mice were anaesthetized with isoflurane (4%; 66% N_2_O and 33% O_2_) and secured in a stereotaxic frame. Anaesthesia was reduced and maintained at 1.5% isoflurane for the remaining duration of the experiment. A laminectomy was performed to expose L3–L5 segments of the spinal cord and extracellular recordings were made from wide dynamic range (WDR) neurons in the deep dorsal horn (lamina III–V, 200–600 mm) using parylene-coated tungsten electrodes (A-M Systems) in *Zfhx2* knockout mice and littermate controls. Mechanical stimuli were applied to the peripheral receptive field of spinal neurons on the hindpaw glabrous skin and the evoked activity of neurons was visualized on an oscilloscope and discriminated on a spike amplitude and waveform basis using a CED 1401 interface coupled to Spike 2 software (Cambridge Electronic Design, UK). Mechanical stimuli (innocuous brush stroke and noxious prods, 100 g/cm^2^ and 150 g/cm^2^) were applied in ascending order of intensity to receptive fields for 10 s and the total number of evoked spikes recorded.

### Microarrays

Following euthanization by inhalation of CO_2_ and cervical dislocation, lumbar DRGs (L1–L6) were isolated from five mice bearing four genomic copies of the mutant *Zfhx2* gene and from seven wild-type controls. Total RNA was isolated using the PureLink^™^ RNA Micro Kit (Invitrogen) and run on the GeneChip Mouse Transcriptome Array 1.0 (Affymetrix). Expression data were analysed using the Expression and Transcriptome Analysis Consoles (Affymetrix). Microarray data have been deposited at Gene Expression Omnibus Array Express for public use with reference number E-MTAB-5650.

### Calcium imaging

Mouse DRG neurons were extracted and dissociated as previously described (Emery *et al.*, 2016), and plated on poly-l-lysine and laminin coated glass coverslips (13 mm; size 0) and cultured in standard cell medium (Dulbecco’s modified Eagle medium + GlutaMAX^™^, Life Technologies) supplemented with 10% foetal bovine serum and nerve growth factor (50 ng/ml). For calcium imaging experiments, neurons were preincubated with 5 µM fluo-4 AM (Life Technologies) in standard extracellular solution (in mM: 124 NaCl, 4 KCl, 10 HEPES, 1 MgCl_2_, 2 CaCl_2_, 5 glucose) for 30 min at 37°C. After the preincubation, neurons were washed in warmed standard extracellular solution and placed into a laminar flow imaging chamber (Warner Instruments). A gravity-fed perfusion system was used to continuously perfuse the neurons throughout the experiment. Image acquisition was performed using a confocal microscope (Leica SP8). For fluo-4 AM excitation, a laser wavelength of 488 nm was used, and the images were acquired at a bidirectional scan speed of 800 Hz. Image analysis was performed using LAS-X software (Leica).

## Results

### Clinical description

**Figure 1:**
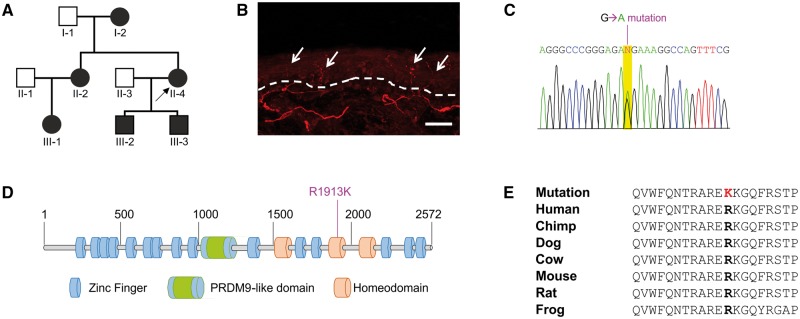
**Missense mutation in ZFHX2 identified in pain insensitive family.** (**A**) Marsili syndrome pedigree showing autosomal dominant inheritance pattern of the pain insensitive phenotype. (**B**) Microphotograph of a skin punch biopsy of the proband (Individual II-4) carrying the mutation. PGP9.5 positive profiles (in red; shown by white arrows) were counted as they crossed the dermal-epidermal junction (dotted line). Using the immunofluorescence method, 11.3 fibres/mm were counted and 6.4 fibres/mm using DAB (not shown). The intraepidermal nerve fibre density (IENFD) count was normal for age and gender. Scale bar = 50 μm. (**C**) Sanger sequencing trace showing the *ZFHX2* mutation (NM_033400:c.G5738A) that was PCR-amplified from genomic DNA isolated from the proband. All affected individuals are heterozygous for this mutation. (**D**) Schematic representation of ZFHX2 protein domain structure with the zinc finger motifs, PRDM9-like domain (aa 1033–1277) and three homeodomains (aa 1595–1657, 1857–1919 and 2065–2127) annotated. The location of the p.R1913K mutation within the second homeodomain is also indicated. See Supplementary Fig. 1 for more information. (**E**) Sequence alignment around mutation site for orthologous *Tetrapoda* ZFHX2 proteins.

We studied a previously described family with a hypoalgesic phenotype in which six affected individuals have a history of painless injuries from childhood (Fig. 1A) (Spinsanti *et al.*, 2008). Bone fractures of the arms and legs are associated with an absence of pain, or pain present only for a few seconds and sometimes accompanied by fainting (Supplementary Table 1). Notably, broken limbs can be used without any painful sensations. The subjects did not record the occurrence of these events immediately and for this reason it is not clear if the time of healing was altered. Burning stimuli are also frequently reported to be not painful, with consequent lesions noticed only by sight. All individuals have severe corneal hyporeflexia but without corneal scarring and with normal tear production. Sweating is scarce or absent in all affected individuals and sensation of warm and cool temperatures is also reported to be altered in five of six subjects, with occasional episodes of hyperthermia (Spinsanti *et al.*, 2008). While cutaneous injuries and bone fractures are often neglected, the pain insensitive phenotype is not global with, for example, lower back pain and occasional headaches promptly and fully perceived. Painful sensations were also noted during childbirth. Sensory phenotype assessments showed that innocuous light touch stimuli could be detected normally (Supplementary Table 1). Heat pain thresholds were variable between tested individuals, with one individual particularly showing extreme insensitivity to noxious heat (Supplementary Table 1). Cold pain thresholds are also variable between individuals, ranging from hyperalgesia in one individual to a very high cold pain threshold and tolerance in another (Supplementary Table 1). The proband (Individual II-4 in Fig. 1A) reported no pain in the mechanical pain threshold detection tests and, interestingly, at high forces a sensation of pleasure was reported (Supplementary Table 1). This is consistent with intense deep pressure from massage being pleasurable, as also noted in a second affected member in the family (Individual III-3 in Fig. 1A). All affected individuals have a low sensitivity to capsaicin as shown by their ability to eat a large amount of hot pepper without any discomfort. In the capsaicin test, carried out on the forearm, the proband reported an immediate and very intense pain following capsaicin injection that, unusually, abruptly (50–60 s) disappeared (Supplementary Table 1). All patients self-report of being able to perceive odours (*SCN9A*-associated pain insensitive individuals are anosmic) although some report a higher than normal sense of smell that is associated with nausea and vomiting. All individuals have normal cognitive abilities, display no evidence of distal weakness nor joint abnormalities and exercise regularly. A skin punch biopsy of the distal leg in the proband followed by measurement of the intraepidermal nerve fibre density showed normal fibre counts for age and gender (11.3 fibres/mm using immunofluorescence) (Provitera *et al.*, 2016), excluding the likelihood of a small fibre neuropathy in the proband (Fig. 1B).

### Whole exome sequencing

The phenotype segregated amongst the six affected individuals within the family in a manner consistent with autosomal dominant inheritance (Fig. 1A). Exome sequencing was performed for all six affected members to identify the pathogenic variant. Following filtering and verification by Sanger sequencing, two novel coding variants were confirmed to co-segregate with the pain-insensitive phenotype: *SUPT3H* (NM_003599:c.G409A:p.A137T) and *ZFHX2* (NM_033400:c.G5738A:p.R1913K). The variant in *SUPT3H* is annotated as benign by both the M-CAP and PolyPhen-2 (HumVar) tools and is also predicted to not alter splicing (NNSPLICE). In contrast, the point mutation in *ZFHX2* (Fig. 1C) was indicated to be deleterious (M-CAP, PolyPhen-2 and SIFT), was absent from public SNP databases (1000 Genomes and dbSNP147) and the ExAC and Institutional (UCL) exome datasets, and so was prioritized for further analysis. *ZFHX2* encodes a large protein of 2572 amino acids (NP_207646) that comprises 17 zinc finger motifs, two of which are adjacent to either side of the central PRDM9-like domain, and three homeodomains (Fig. 1D and Supplementary Fig. 1), features common to transcriptional regulators (Komine *et al.*, 2006). The predicted PRDM9-like fold domain has no common active site tyrosines essential for methyl transferase activity (Wu *et al.*, 2013) but may still exhibit nucleosome binding properties. The p.R1913K mutation identified in the pain-insensitive individuals maps within the second homeodomain (Fig. 1D) and to a residue that is invariant across all known orthologous *Tetrapoda Zfhx2* genes (HomoloGene 52657) (Fig. 1E).

### *Zfhx2* knockout mice have altered pain thresholds

**Figure 2:**
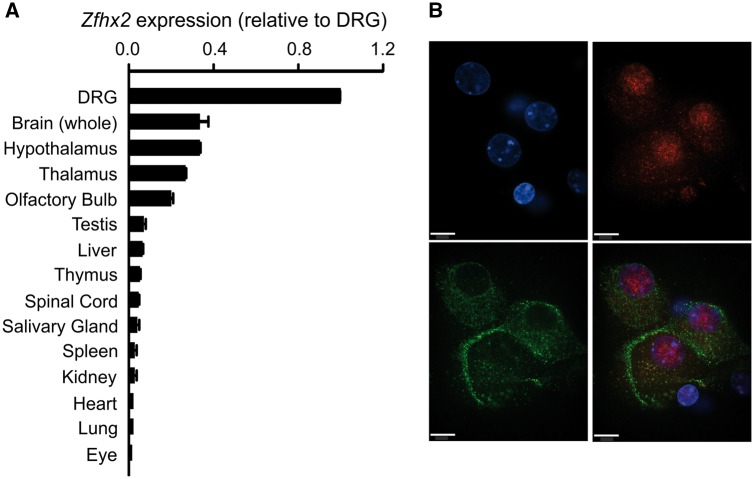
***Zfhx2* is highly expressed in DRG within peripherin-positive neurons.** (**A**) Real-time qPCR assay measuring the expression level of *Zfhx2* in specific tissues from adult wild-type mice (*n = *3). (**B**) Immunocytochemical analysis of PFA fixed dissociated DRG derived from an adult C57BL/6 wild-type mouse. DAPI staining (in blue), rabbit polyclonal anti-ZFHX2 (in red), mouse monoclonal anti-peripherin (in green). ZFHX2 localizes to the nucleus of peripherin-positive small diameter DRG neurons. Scale bar = 10 μm.

**Figure 3:**
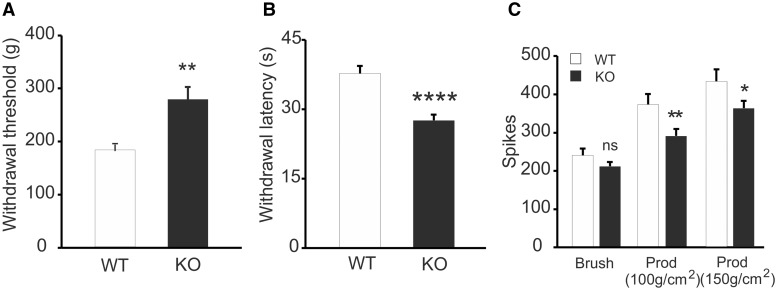
**Zfhx2 knockout mice have altered acute mechanical and thermal pain thresholds.** (**A**) Randall Selitto test measuring withdrawal thresholds in the tail to noxious mechanical stimuli in knockout (KO; *n = *15) and wild-type (WT) littermates (*n = *13). Knockout mice are significantly hyposensitive compared to controls (*P* = 0.0017). (**B**) Hot plate test measuring withdrawal latency to noxious thermal (50°C) stimuli in knockout (*n = *31) and wild-type littermate (*n = *31) controls. Knockout mice are significantly hypersensitive compared to controls (*P* = 0.000008). (**C**) ZFHX2 knockout deep dorsal horn WDR neurons show a deficit in noxious mechanical coding compared to littermate controls but no differences in response to dynamic low threshold stimuli. Brush (wild-type *n = *44, knockout *n = *61, *P* = 0.176); Prod 100 g/cm^2^ (wild-type *n = *43, knockout *n = *61, *P* = 0.01); Prod 150 g/cm^2^ (wild-type *n = *43, knockout *n = *61, *P* = 0.044). All data analysed by *t*-test. Results are presented as mean ± standard error of the mean (SEM); ns (not significant) *P* > 0.05; *P ≤ 0.05; **P ≤ 0.01; ****P ≤ 0.0001.

*Zfhx2* was cloned in 2006 and a global knockout mouse was reported in 2012, showing several behavioural abnormalities, namely, hyperactivity, enhanced depression-like behaviours, and an aberrantly altered anxiety-like phenotype (Komine *et al.*, 2006, 2012). *Zfhx2* was shown to be highly expressed in the developing brain, including the thalamus, hypothalamus, midbrain and pontine areas, with expression persisting in adult brain. Using RT-PCR in adult wild-type mice, we found that *Zfhx2* expression is enriched within DRGs (Fig. 2A) and immunocytochemical analyses showed nuclear staining of ZFHX2 within peripherin-positive small diameter neurons (Fig. 2B). We investigated whether *Zfhx2* global knockout mice display an acute pain behavioural phenotype using a battery of assays designed to test thermal and mechanical acute pain thresholds (see ‘Materials and methods’ section, Fig. 3A, B and Supplementary Fig. 2A–D). These showed that the global knockout mice, compared to their wild-type littermates, were significantly hyposensitive to noxious mechanical stimuli applied to the tail but with normal sensitivity to innocuous touch (Fig. 3A and Supplementary Fig. 2B). These data are supported by deep dorsal horn WDR neuron recordings in *Zfhx2* knockouts, which showed a significant deficit in noxious mechanical coding compared to littermate controls but no differences in response to dynamic low threshold stimuli (brush) (Fig. 3C). Interestingly, in the hot-plate test the *Zfhx2* knockout animals were significantly hypersensitive to noxious heat (Fig. 3B), highlighting the importance of the nociceptor-expressed *Zfhx2* gene in regulating both mechanical and thermal acute pain thresholds.

### ZFHX2 p.R1907K transgenic mice are hyposensitive to noxious heat

**Figure 4:**
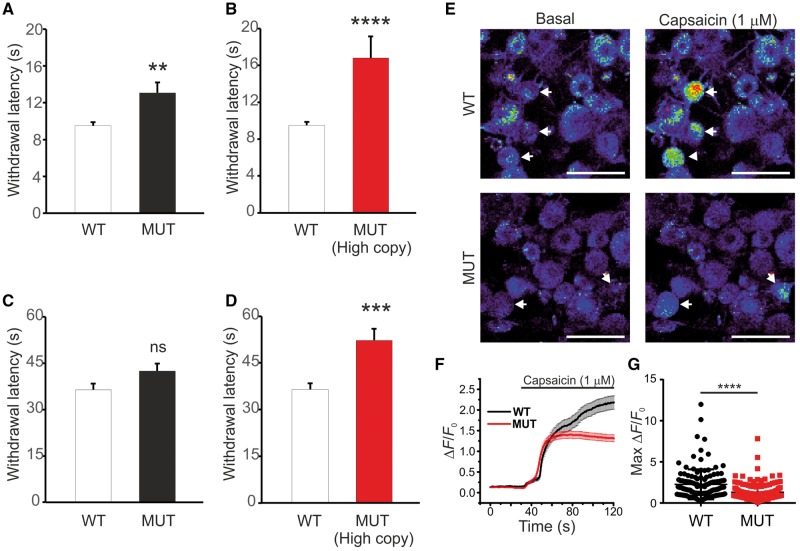
**ZFHX2 p.R1907K BAC transgenic mice are hyposensitive to noxious heat and DRG neurons have reduced responses to capsaicin.** (**A**) Hargreaves’ test measuring paw withdrawal latency to noxious thermal stimuli. Wild-type (WT) *n = *16, mutant (MUT) *n = *16 (genomic BAC copy number 1–5), *P* = 0.0048. (**B**) Mice with the highest genomic BAC copy number (four to five copies) have a high pain threshold to noxious heat. Wild-type *n = *16, mutant *n = *5, *P* = 0.000058. (**C**) Hot-plate test measuring withdrawal latency to noxious thermal (50°C) stimuli in BAC transgenic mice (mutant *n = *16) and wild-type littermate (*n = *16) controls. *P* = 0.058. (**D**) Similar to the Hargreaves’ test, mice with the highest genomic BAC copy number (four to five copies) have a high pain threshold to noxious heat. Wild-type *n = *16, mutant *n = *5, *P* = 0.00095. (**E**) Example confocal images from cultured wild-type and mutant mouse DRG neurons before and after the application of capsaicin (1 µM). Scale bar = 50 µm. (**F**) Averaged response of all wild-type (*n = *129) and mutant (*n = *138) mouse DRG neurons to capsaicin (1 µM) application. (**G**) Maximal relative fluorescence from baseline for wild-type and mutant mouse DRG neurons following capsaicin (1 µM) application. *P* = 0.0000002. All data analysed by *t-*test. Results are presented as mean ± SEM; ns *P* > 0.05; **P ≤ 0.01; ***P ≤ 0.001; ****P ≤ 0.0001.

To investigate the effects of the p.R1913K ZFHX2 missense mutation we cloned the full-length coding sequence of the human gene (GenBank KY781180) and expressed the wild-type (R1913) and the mutant (K1913) versions in AD293 cells (Supplementary material). Both the wild-type and mutant ZFHX2 proteins localized to the nucleus in transiently transfected cells, indicating that the phenotype was unlikely to be caused by a subcellular trafficking defect (Supplementary Fig. 3). Next, a BAC transgenic mouse model was generated in which arginine-1907, the orthologous amino acid to arginine-1913 in human ZFHX2, was mutated to lysine. Acute pain behaviour assays (Fig. 4 and Supplementary Fig. 4) showed that transgenic mice expressing the ZFHX2 p.R1907K mutant protein had significant deficits in sensitivity to noxious heat. Strikingly, the noxious heat pain thresholds were significantly higher in mice with the highest genomic BAC copy number (four to five genomic copies) (Fig. 4B and D). Furthermore, calcium imaging experiments conducted on cultured DRG neurons from these mice showed that they had a reduced response to capsaicin (1 µM) compared to wild-type neurons (Fig. 4E–G). Immunohistochemical analysis of lumbar (L4) DRGs isolated from adult BAC transgenic mice showed no significant differences in the number of peripherin-positive neurons compared to wild-type controls, indicating that overexpression of mutant ZFHX2 was not associated with a loss of small diameter nociceptive neurons (Supplementary Fig. 5).

### Significant gene expression deregulation in dorsal root ganglion neurons from mutant mice

**Figure 5:**
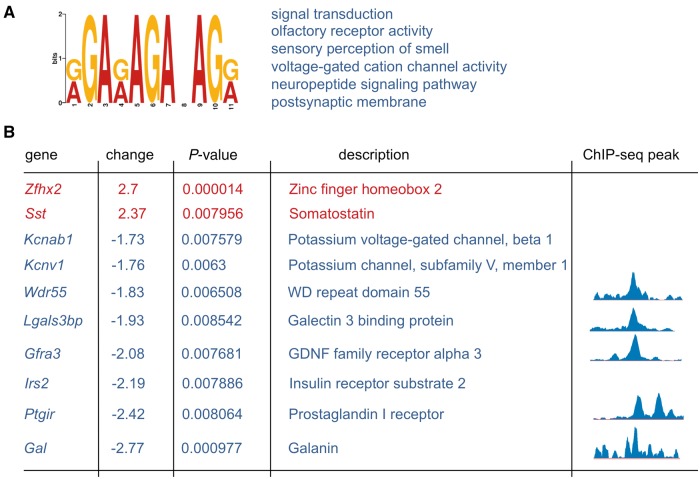
**Potential transcriptional targets of ZFHX2.** (**A**) The consensus AG-rich motif derived from promoter regions of deregulated genes in the microarray screen. The motif has been constructed from five common motifs highly represented in 1000-bp upstream regions (Supplementary Fig. 6). (**B**) The top six GO terms for genes that contain the AG-rich motif. For more information see Supplementary Table 4. The genes with the biggest change in their expression levels (*P* < 0.01). *Zfhx2* expression level is shown as a positive control as it is expected to rise after introducing extra copies of the *Zfhx2*^mut^ gene. Some of these genes have shown an enrichment of ZFHX2 binding at their regulatory upstream regions (as indicated in *right* column) as analysed by ChIP-seq analysis.

ZFHX2 is a putative transcriptional regulator and so we studied the effects of the p.R1907K mutation on gene expression profiles in mice bearing four genomic copies of the mutant *Zfhx2* gene. Lumbar DRGs (L1–L6) were isolated from mutant and wild-type controls and RNA analysed using microarrays (Fig. 5 and Supplementary Table 2). Using an ANOVA *P*-value cut-off of ≤0.01, six genes were upregulated more than 1.7-fold in mutant DRGs versus controls. Sixteen genes were downregulated more than 1.7-fold in mutant DRGs versus controls (Supplementary Table 2). Interestingly a number of genes in this list such as *Gal*, *Sst*, *Gfra3*, *Ptgir* and others are known for their connection to pain signalling. To explore whether the genes deregulated in DRGs as a result of mutant *Zfhx2* expression share any DNA sequence motifs in common we performed a phylogenetic shadowing-based approach (Bailey and Elkan, 1994) using promoter region sequences from genes which showed at least a 1.2-fold expression level change with ANOVA *P* ≤ 0.01 (Supplementary material and Supplementary Table 2). Analysis of these genes yielded four common AG-rich DNA sequence motifs (Supplementary Fig. 6B) present in ∼60% of 119 analysed genes. To verify that these AG-rich motifs are present in ZFHX2 DNA-binding regions we used human neuroblastoma SH-SY5Y cells expressing V5-tagged mutant ZFHX2 for ChIP-seq analysis (Supplementary material). Forty-five genes of those 119 being shown to be significantly deregulated in the microarray screen showed ChIP-seq peaks within their immediate promoter regions, which were different to the negative controls (IgG ChIP) and had AG-rich motifs present within immunoprecipitated DNA sequences (Supplementary Table 3 and Supplementary Fig. 6C). Among those genes were *Gfra3*, *Gal* and *Ptgir* (Fig. 5B). One AG-rich motif from those sequences (Supplementary Fig. 6B) was aligned with four earlier identified AG-rich motifs from genes deregulated in microarray experiments to generate the AG-rich consensus motif with STAMP software (Fig. 5A and Supplementary Fig. 6A). The gene ontology search for the resulting AG-rich motif showed its presence in gene families functionally engaged in signal transduction, neuropeptide signalling and voltage-gated cation channel activity (Fig. 5A and Supplementary Table 4). Interestingly, the motif was also present in genes engaged in regulation of olfactory receptor activity and sensory perception of smell. The differentially expressed genes highlighted here, either alone or in combination, potentially contribute to the pain insensitive phenotype.

## Discussion

Chronic pain remains a severe clinical problem affecting billions of people worldwide and despite the intense efforts of the pharmaceutical industry, it is still poorly treated in a significant number of patients (Nahin, 2015). The study of families with monogenic pain insensitivity disorders remains a powerful route to identify novel analgesic drug targets (Goldberg *et al.*, 2012). For example, since the discovery of the first *SCN9A* loss of function mutations in 2006, significant progress has been made to help understand why loss of function of Na_v_1.7 leads to complete analgesia (Minett *et al.*, 2015). This work is informing drug development strategies on how to better reproduce this phenotype pharmacologically, such as through the combination of Na_v_1.7 channel blockers with low dose opioids or enkephalinase inhibitors (Minett *et al.*, 2015; Deuis *et al.*, 2017). NGF and TRK-A, previously shown to be essential for the development of nociceptors, have also since been realized to be useful analgesic targets with anti-NGF drugs already showing great promise in clinical trials (Holmes, 2012). The analgesic action of anti-NGFs is related to the post-developmental importance of NGF in sensitizing nociceptors and thereby increasing the response to noxious stimuli (Hirose *et al.*, 2016).

Here we have studied a family with six members affected with a pain insensitive phenotype, characterized by multiple painless bone fractures and frequent painless lesions caused by burning stimuli. Notwithstanding these injuries, all subjects carried out a normal life and only occasionally had the faint perception that something was different. It was called Marsili syndrome, from the name of the family. Using exome sequencing, we identified a novel coding mutation in the *ZFHX2* gene, which alters a strictly conserved arginine to a lysine residue within the second homeodomain. The full-length ZFHX2 protein contains three homeodomains, DNA-binding regions typically 60 amino acids long, in addition to 17 zinc-finger motifs and a PRDM9-like domain, which is probably involved in nucleosome/histone binding, indicating that ZFHX2 is likely to be a potent transcriptional regulator. The mutated arginine is located at position 57 within the homeodomain. In the consensus homeodomain sequence, lysine-57 contacts with the DNA backbone (Gehring *et al.*, 1994; D’Elia *et al.*, 2001). Although position 57 is often interchangeable between arginine and lysine in different homeodomain-containing proteins, arginine-1913 in ZFHX2 is evolutionarily conserved in every sequenced orthologue on the UCSC genome browser, highlighting the likely functional importance of this residue. How this mutation alters the function of ZFHX2 is still to be clarified, but potentially it alters DNA binding affinity and/or transcriptional specificity of mutant ZFHX2, which could involve a post-translational modification at the mutation site and/or altered protein-protein interactions with transcriptional partners (Bobola and Merabet, 2017).

We have shown that the missense mutation in ZFHX2 causes a pain insensitive phenotype in humans and in the BAC transgenic mouse model, with both displaying deficits in responses to acute noxious heat. Furthermore, the affected family members are hyposensitive to capsaicin (Supplementary Table 1), which is consistent with the reduced responses recorded by calcium imaging in DRG neurons isolated from BAC transgenic mice (Fig. 4E–G). Interestingly, the *Zfhx2* null mouse is hypersensitive to noxious heat and shows a deficit in the Randall-Selitto test for noxious mechanical pain, linking the expression of the transcription factor in sensory neurons with altered nociceptive processing. However, the BAC transgenic mouse failed to exhibit deficits in the noxious mechanical pain test or in the cold plantar assay for noxious cold, indicating that the human phenotype is not fully recapitulated by this particular mouse model. Furthermore, the human individuals have additional symptoms that we have yet to confirm in the transgenic mutant mice, such as painless bone fractures, reduced sweating and pleasure upon intense mechanical pressure. The differences in the human and mouse phenotypes could be related to man–mouse differences in ZFHX2 functions. Another explanation is that in the transgenic mouse, two wild-type alleles still exist that could be reducing the effect of expressing the mutant ZFHX2 protein. This is a likely possibility since we observed that the heat-insensitive phenotype in the Hargreaves’ and hot-plate tests is enhanced in animals with more genomic copies of the mutant gene (Fig. 4B and D). However, we cannot exclude the possibility that the observed phenotype in the BAC transgenic line is due to overexpression of ZFHX2. Generation and analysis of a ZFHX2 p.R1907K knockin mouse model and further behaviour tests should help to clarify the full extent of the mouse phenotype. This will include understanding the variability in severity of symptoms, a feature noted in the human family and which is not uncommon in other dominantly inherited disorders.

ZFHX2 has enriched expression within DRGs, particularly within the peripherin positive population of neurons, and so we investigated if the pain insensitive phenotype was due to a peripheral neuropathy. However, the intraepidermal nerve fibre density in a punch skin biopsy from the proband and DRG cell counts in the BAC transgenic mice were within normal ranges. Given the role of ZFHX2 as a putative transcriptional regulator, we therefore assessed the gene expression profiles in whole dorsal root ganglia of BAC transgenic and wild-type animals to see if critical genes within nociceptive neurons were affected by the mutation. Several genes previously implicated in peripheral pain mechanisms were differentially expressed (Fig. 5 and Supplementary Table 2). For example, somatostatin, which is upregulated >2-fold in the DRGs from the mutant mice, has proven anti-nociceptive and anti-inflammatory effects in experimental animals and can relieve pain in humans (Mollenholt *et al.*, 1994; Carlton *et al.*, 2001; Helyes *et al.*, 2004; Pinter *et al.*, 2006). Likewise, prostacyclin is an important mediator of inflammation and pain (Bley *et al.*, 1998) and the mutant *Zfhx2* mice have a 2.4-fold downregulation of the prostacyclin receptor, PTGIR. Antagonism of this receptor and work in PTGIR-deficient mice have shown reduced pain and inflammation in various rodent models of acute and inflammatory pain (Murata *et al.*, 1997; Pulichino *et al.*, 2006). Another gene significantly downregulated in mutant DRG is *Gfra3* (GDNF receptor alpha 3), a gene that when knocked out in mice, results in hyposensitivity to noxious thermal stimuli in the Hargreaves’ and tail-flick tests (Murota *et al.*, 2012). Galanin (*Gal*), the gene most downregulated in mutant DRGs, plays a complex role in pain signalling, with both facilitatory and inhibitory effects on nociceptive processing (Holmes *et al.*, 2003). Which of the above genes or any of the other less characterized DRG-expressed genes (e.g. *Fam89a*) are principally responsible for the pain-insensitive phenotype remains to be determined. As the microarrays reflect an average snapshot of multiple types of neurons within DRG, some genes may be deregulated more significantly in specific types of neurons. Thus, although we see strong changes for a group of genes we cannot exclude that some other interesting and important genes also contribute to the phenotype despite low fold changes in the current analysis of the whole DRG. Gene expression analyses in whole DRG and specific neuron populations within the DRG, and perhaps in brain regions such as the thalamus, in a knockin mouse model should help us focus on potential downstream analgesic drug targets. It remains possible that the pain insensitivity results from a combined effect of deregulating several genes, and in this scenario the mutant *ZFHX2* gene itself, perhaps via intrathecal AAV delivery, could offer a potential route for providing pain relief.

The microarray analyses were complemented by ChIP-seq experiments in human neuroblastoma cells stably expressing the mutant ZFHX2 protein. These experiments identified a likely consensus genomic binding motif for ZFHX2 that was present upstream of several of the deregulated genes identified in the DRG microarray (including *Ptgir*, *Gal* and *Gfra3*). Interestingly, gene ontology searches showed this AG-rich motif was present in gene families functionally engaged in neuropeptide signalling. Similarly, a dominant point mutation in the homologous *Zfhx3* gene was also shown to alter neuropeptide gene expression, in the suprachiasmatic nucleus, in a mouse model with accelerated circadian locomotor rhythms (Parsons *et al.*, 2015). Furthermore, the gene ontology searches for the ZFHX2 AG-rich motif also identified genes engaged in regulation of olfactory receptor activity and sensory perception of smell. Given this and the known expression of *Zfhx2* in the olfactory bulb (Fig. 2A and Allen Brain Atlas), together with the heightened sense of smell reported in some members of the human family, it is likely that mutant ZFHX2 may be important for olfaction as well as nociceptive processing. An analogy here is Na_v_1.7, which has a critical role in nociceptive and olfactory signalling with recessive loss of function mutations causing pain insensitivity and anosmia (Cox *et al.*, 2006; Weiss *et al.*, 2011). Importantly, there is no apparent link between other genes implicated in heritable loss of pain such as *NTRK1/*TRK-A, *NGF* or *SCN9A* and the deregulated genes found in the *ZFHX2* microarray analysis. This suggests an entirely novel mechanism is involved in conferring a pain-insensitive phenotype in the family studied here.

In summary, genetic analysis of a human family with Marsili syndrome, a rare and perhaps unique inherited pain insensitive phenotype, and mouse modelling have shown *ZFHX2* as a critical gene for normal pain perception. Further work will resolve how the deregulated DRG-expressed genes contribute to the hypoalgesic phenotype and will help to determine which ones are feasible analgesic drug targets that could lead to better treatments for chronic pain.

## Supplementary Material

Supplementary Figures

Supplemental Materials and Methods

Supplementary Table S1

Supplementary Data

Supplementary Data

Supplementary Data

Supplementary Data

## Abbreviations

BAC: bacterial artificial chromosome
DRG: dorsal root ganglia
HSAN: hereditary sensory and autonomic neuropathy

## References

[awx326-B1] Bailey TL, Elkan C Fitting a mixture model by expectation maximization to discover motifs in biopolymers. Proc Int Conf Intell Syst Mol Biol 1994; 2: 28–36. 7584402

[awx326-B2] Bley KR, Hunter JC, Eglen RM, Smith JA The role of IP prostanoid receptors in inflammatory pain. Trends Pharmacol Sci 1998; 19: 141–7. 9612089 10.1016/s0165-6147(98)01185-7

[awx326-B3] Bobola N, Merabet S Homeodomain proteins in action: similar DNA binding preferences, highly variable connectivity. Curr Opin Genet Dev 2017; 43: 1–8. 27768937 10.1016/j.gde.2016.09.008

[awx326-B4] Brenner DS, Golden JP, Gereau RW IV A novel behavioral assay for measuring cold sensation in mice. PLoS One 2012; 7: e39765. 22745825 10.1371/journal.pone.0039765 PMC3382130

[awx326-B5] Carlton SM, Du J, Zhou S, Coggeshall RE Tonic control of peripheral cutaneous nociceptors by somatostatin receptors. J Neurosci 2001; 21: 4042–9. 11356891 10.1523/JNEUROSCI.21-11-04042.2001 PMC6762714

[awx326-B6] Carvalho OP, Thornton GK, Hertecant J, Houlden H, Nicholas AK, Cox JJ, et al A novel NGF mutation clarifies the molecular mechanism and extends the phenotypic spectrum of the HSAN5 neuropathy. J Med Genet 2011; 48: 131–5. 20978020 10.1136/jmg.2010.081455 PMC3030776

[awx326-B7] Chaplan SR, Bach FW, Pogrel JW, Chung JM, Yaksh TL Quantitative assessment of tactile allodynia in the rat paw. J Neurosci Methods 1994; 53: 55–63. 7990513 10.1016/0165-0270(94)90144-9

[awx326-B8] Chen YC, Auer-Grumbach M, Matsukawa S, Zitzelsberger M, Themistocleous AC, Strom TM, et al Transcriptional regulator PRDM12 is essential for human pain perception. Nat Genet 2015; 47: 803–8. 26005867 10.1038/ng.3308 PMC7212047

[awx326-B9] Cox JJ, Reimann F, Nicholas AK, Thornton G, Roberts E, Springell K, et al An SCN9A channelopathy causes congenital inability to experience pain. Nature 2006; 444: 894–8. 17167479 10.1038/nature05413 PMC7212082

[awx326-B10] D'Elia AV, Tell G, Paron I, Pellizzari L, Lonigro R, Damante G Missense mutations of human homeoboxes: a review. Hum Mutat 2001; 18: 361–74. 11668629 10.1002/humu.1207

[awx326-B11] DePristo MA, Banks E, Poplin R, Garimella KV, Maguire JR, Hartl C, et al A framework for variation discovery and genotyping using next-generation DNA sequencing data. Nat Genet 2011; 43: 491–8. 21478889 10.1038/ng.806 PMC3083463

[awx326-B12] Deuis JR, Dekan Z, Wingerd JS, Smith JJ, Munasinghe NR, Bhola RF, et al Pharmacological characterisation of the highly NaV1.7 selective spider venom peptide Pn3a. Sci Rep 2017; 7: 40883. 28106092 10.1038/srep40883 PMC5247677

[awx326-B13] Eddy NB, Leimbach D Synthetic analgesics. II. Dithienylbutenyl- and dithienylbutylamines. J Pharmacol Exp Ther 1953; 107: 385–93. 13035677

[awx326-B14] Einarsdottir E, Carlsson A, Minde J, Toolanen G, Svensson O, Solders G, et al A mutation in the nerve growth factor beta gene (NGFB) causes loss of pain perception. Hum Mol Genet 2004; 13: 799–805. 14976160 10.1093/hmg/ddh096

[awx326-B15] Emery EC, Luiz AP, Sikandar S, Magnusdottir R, Dong X, Wood JN *In vivo* characterization of distinct modality-specific subsets of somatosensory neurons using GCaMP. Sci Adv 2016; 2: e1600990. 27847865 10.1126/sciadv.1600990 PMC5106201

[awx326-B16] Gehring WJ, Qian YQ, Billeter M, Furukubo-Tokunaga K, Schier AF, Resendez-Perez D, et al Homeodomain-DNA recognition. Cell 1994; 78: 211–23. 8044836 10.1016/0092-8674(94)90292-5

[awx326-B17] Goldberg YP, Pimstone SN, Namdari R, Price N, Cohen C, Sherrington RP, et al Human Mendelian pain disorders: a key to discovery and validation of novel analgesics. Clin Genet 2012; 82: 367–73. 22845492 10.1111/j.1399-0004.2012.01942.x

[awx326-B18] Habib AM, Wood JN, Cox JJ Sodium channels and pain. Handb Exp Pharmacol 2015; 227: 39–56. 25846613 10.1007/978-3-662-46450-2_3

[awx326-B19] Hargreaves K, Dubner R, Brown F, Flores C, Joris J A new and sensitive method for measuring thermal nociception in cutaneous hyperalgesia. Pain 1988; 32: 77–88. 3340425 10.1016/0304-3959(88)90026-7

[awx326-B20] Helyes Z, Szabo A, Nemeth J, Jakab B, Pinter E, Banvolgyi A, et al Antiinflammatory and analgesic effects of somatostatin released from capsaicin-sensitive sensory nerve terminals in a Freund's adjuvant-induced chronic arthritis model in the rat. Arthritis Rheum 2004; 50: 1677–85. 15146439 10.1002/art.20184

[awx326-B21] Hirose M, Kuroda Y, Murata E NGF/TrkA signaling as a therapeutic target for pain. Pain Pract 2016; 16: 175–82. 26452158 10.1111/papr.12342

[awx326-B22] Holmes D Anti-NGF painkillers back on track? Nat Rev Drug Discov 2012; 11: 337–8. 22543456 10.1038/nrd3732

[awx326-B23] Holmes FE, Bacon A, Pope RJ, Vanderplank PA, Kerr NC, Sukumaran M, et al Transgenic overexpression of galanin in the dorsal root ganglia modulates pain-related behavior. Proc Natl Acad Sci USA 2003; 100: 6180–5. 12721371 10.1073/pnas.0937087100 PMC156346

[awx326-B24] Indo Y, Tsuruta M, Hayashida Y, Karim MA, Ohta K, Kawano T, et al Mutations in the TRKA/NGF receptor gene in patients with congenital insensitivity to pain with anhidrosis. Nat Genet 1996; 13: 485–8. 8696348 10.1038/ng0896-485

[awx326-B25] Komine Y, Nakamura K, Katsuki M, Yamamori T Novel transcription factor zfh-5 is negatively regulated by its own antisense RNA in mouse brain. Mol Cell Neurosci 2006; 31: 273–83. 16257534 10.1016/j.mcn.2005.09.017

[awx326-B26] Komine Y, Takao K, Miyakawa T, Yamamori T Behavioral abnormalities observed in Zfhx2-deficient mice. PLoS One 2012; 7: e53114. 23300874 10.1371/journal.pone.0053114 PMC3534046

[awx326-B27] Lauria G, Hsieh ST, Johansson O, Kennedy WR, Leger JM, Mellgren SI, et al European Federation of Neurological Societies/Peripheral Nerve Society Guideline on the use of skin biopsy in the diagnosis of small fiber neuropathy. Report of a joint task force of the European Federation of Neurological Societies and the Peripheral Nerve Society. Eur J Neurol 2010; 17: 903–12, e44–9. 20642627 10.1111/j.1468-1331.2010.03023.x

[awx326-B28] Leipold E, Liebmann L, Korenke GC, Heinrich T, Giesselmann S, Baets J, et al A *de novo* gain-of-function mutation in SCN11A causes loss of pain perception. Nat Genet 2013; 45: 1399–404. 24036948 10.1038/ng.2767

[awx326-B29] Li H, Durbin R Fast and accurate short read alignment with Burrows-Wheeler transform. Bioinformatics 2009; 25: 1754–60. 19451168 10.1093/bioinformatics/btp324 PMC2705234

[awx326-B30] McKenna A, Hanna M, Banks E, Sivachenko A, Cibulskis K, Kernytsky A, et al The genome analysis toolkit: a MapReduce framework for analyzing next-generation DNA sequencing data. Genome Res 2010; 20: 1297–303. 20644199 10.1101/gr.107524.110 PMC2928508

[awx326-B31] Minett MS, Pereira V, Sikandar S, Matsuyama A, Lolignier S, Kanellopoulos AH, et al Endogenous opioids contribute to insensitivity to pain in humans and mice lacking sodium channel Na(v)1.7. Nat Commun 2015; 6: 8967. 26634308 10.1038/ncomms9967 PMC4686868

[awx326-B32] Minett MS, Quick K, Wood JN Behavioral measures of pain thresholds. Curr Protoc Mouse Biol 2011; 1: 383–412. 26068997 10.1002/9780470942390.mo110116

[awx326-B33] Mollenholt P, Rawal N, Gordh T Jr, Olsson Y Intrathecal and epidural somatostatin for patients with cancer. Analgesic effects and postmortem neuropathologic investigations of spinal cord and nerve roots. Anesthesiology 1994; 81: 534–42. 7916546 10.1097/00000542-199409000-00004

[awx326-B34] Murata T, Ushikubi F, Matsuoka T, Hirata M, Yamasaki A, Sugimoto Y, et al Altered pain perception and inflammatory response in mice lacking prostacyclin receptor. Nature 1997; 388: 678–82. 9262402 10.1038/41780

[awx326-B35] Murota H, Izumi M, Abd El-Latif MI, Nishioka M, Terao M, Tani M, et al Artemin causes hypersensitivity to warm sensation, mimicking warmth-provoked pruritus in atopic dermatitis. J Allergy Clin Immunol 2012; 130: 671–82.e4. 22770266 10.1016/j.jaci.2012.05.027

[awx326-B36] Nahin RL Estimates of pain prevalence and severity in adults: United States, 2012. J Pain 2015; 16: 769–80. 26028573 10.1016/j.jpain.2015.05.002 PMC4562413

[awx326-B37] Nahorski MS, Chen YC, Woods CG New Mendelian disorders of painlessness. Trends Neurosci 2015; 38: 712–24. 26549885 10.1016/j.tins.2015.08.010

[awx326-B38] Parsons MJ, Brancaccio M, Sethi S, Maywood ES, Satija R, Edwards JK, et al The regulatory factor ZFHX3 modifies circadian function in SCN via an AT Motif-Driven Axis. Cell 2015; 162: 607–21. 26232227 10.1016/j.cell.2015.06.060 PMC4537516

[awx326-B39] Phatarakijnirund V, Mumm S, McAlister WH, Novack DV, Wenkert D, Clements KL, et al Congenital insensitivity to pain: fracturing without apparent skeletal pathobiology caused by an autosomal dominant, second mutation in SCN11A encoding voltage-gated sodium channel 1.9. Bone 2016; 84: 289–98. 26746779 10.1016/j.bone.2015.11.022 PMC4755825

[awx326-B40] Pinter E, Helyes Z, Szolcsanyi J Inhibitory effect of somatostatin on inflammation and nociception. Pharmacol Ther 2006; 112: 440–56. 16764934 10.1016/j.pharmthera.2006.04.010

[awx326-B41] Provitera V, Gibbons CH, Wendelschafer-Crabb G, Donadio V, Vitale DF, Stancanelli A, et al A multi-center, multinational age- and gender-adjusted normative dataset for immunofluorescent intraepidermal nerve fiber density at the distal leg. Eur J Neurol 2016; 23: 333–8. 26493160 10.1111/ene.12842

[awx326-B42] Pulichino AM, Rowland S, Wu T, Clark P, Xu D, Mathieu MC, et al Prostacyclin antagonism reduces pain and inflammation in rodent models of hyperalgesia and chronic arthritis. J Pharmacol Exp Ther 2006; 319: 1043–50. 16973887 10.1124/jpet.106.110387

[awx326-B43] Randall LO, Selitto JJ A method for measurement of analgesic activity on inflamed tissue. Arch Int Pharmacodyn Ther 1957; 111: 409–19. 13471093

[awx326-B44] Rolke R, Baron R, Maier C, Tolle TR, Treede RD, Beyer A, et al Quantitative sensory testing in the German Research Network on Neuropathic Pain (DFNS): standardized protocol and reference values. Pain 2006; 123: 231–43. 16697110 10.1016/j.pain.2006.01.041

[awx326-B45] Rotthier A, Baets J, Timmerman V, Janssens K Mechanisms of disease in hereditary sensory and autonomic neuropathies. Nat Rev Neurol 2012; 8: 73–85. 22270030 10.1038/nrneurol.2011.227

[awx326-B46] Spinsanti G, Zannolli R, Panti C, Ceccarelli I, Marsili L, Bachiocco V, et al Quantitative real-time PCR detection of TRPV1-4 gene expression in human leukocytes from healthy and hyposensitive subjects. Mol Pain 2008; 4: 51. 18983665 10.1186/1744-8069-4-51 PMC2588574

[awx326-B47] Stirling LC, Forlani G, Baker MD, Wood JN, Matthews EA, Dickenson AH, et al Nociceptor-specific gene deletion using heterozygous NaV1.8-Cre recombinase mice. Pain 2005; 113: 27–36. 15621361 10.1016/j.pain.2004.08.015

[awx326-B48] Weiss J, Pyrski M, Jacobi E, Bufe B, Willnecker V, Schick B, et al Loss-of-function mutations in sodium channel Nav1.7 cause anosmia. Nature 2011; 472: 186–90. 21441906 10.1038/nature09975 PMC3674497

[awx326-B49] Wolf S, Hardy JD Studies on pain. Observations on pain due to local cooling and on factors involved in the “cold pressor” effect. J Clin Invest 1941; 20: 521–33. 16694857 10.1172/JCI101245 PMC435082

[awx326-B50] Wolfe F, Smythe HA, Yunus MB, Bennett RM, Bombardier C, Goldenberg DL, et al The American College of Rheumatology 1990 criteria for the classification of fibromyalgia. Report of the Multicenter Criteria Committee. Arthritis Rheum 1990; 33: 160–72. 2306288 10.1002/art.1780330203

[awx326-B51] Woods CG, Babiker MO, Horrocks I, Tolmie J, Kurth I The phenotype of congenital insensitivity to pain due to the NaV1.9 variant p.L811P. Eur J Hum Genet 2015; 23: 561–3. 25118027 10.1038/ejhg.2014.166 PMC4402639

[awx326-B52] Wu H, Mathioudakis N, Diagouraga B, Dong A, Dombrovski L, Baudat F, et al Molecular basis for the regulation of the H3K4 methyltransferase activity of PRDM9. Cell Rep 2013; 5: 13–20. 24095733 10.1016/j.celrep.2013.08.035

