# Supplementary figures and images for: A novel human pain insensitivity disorder caused by a point mutation in ZFHX2
Source: Brain. 2017 Dec 14;141(2):365–76. doi: 10.1093/brain/awx326 (PMC5837393; doi:10.1093/brain/awx326)

Figure S2

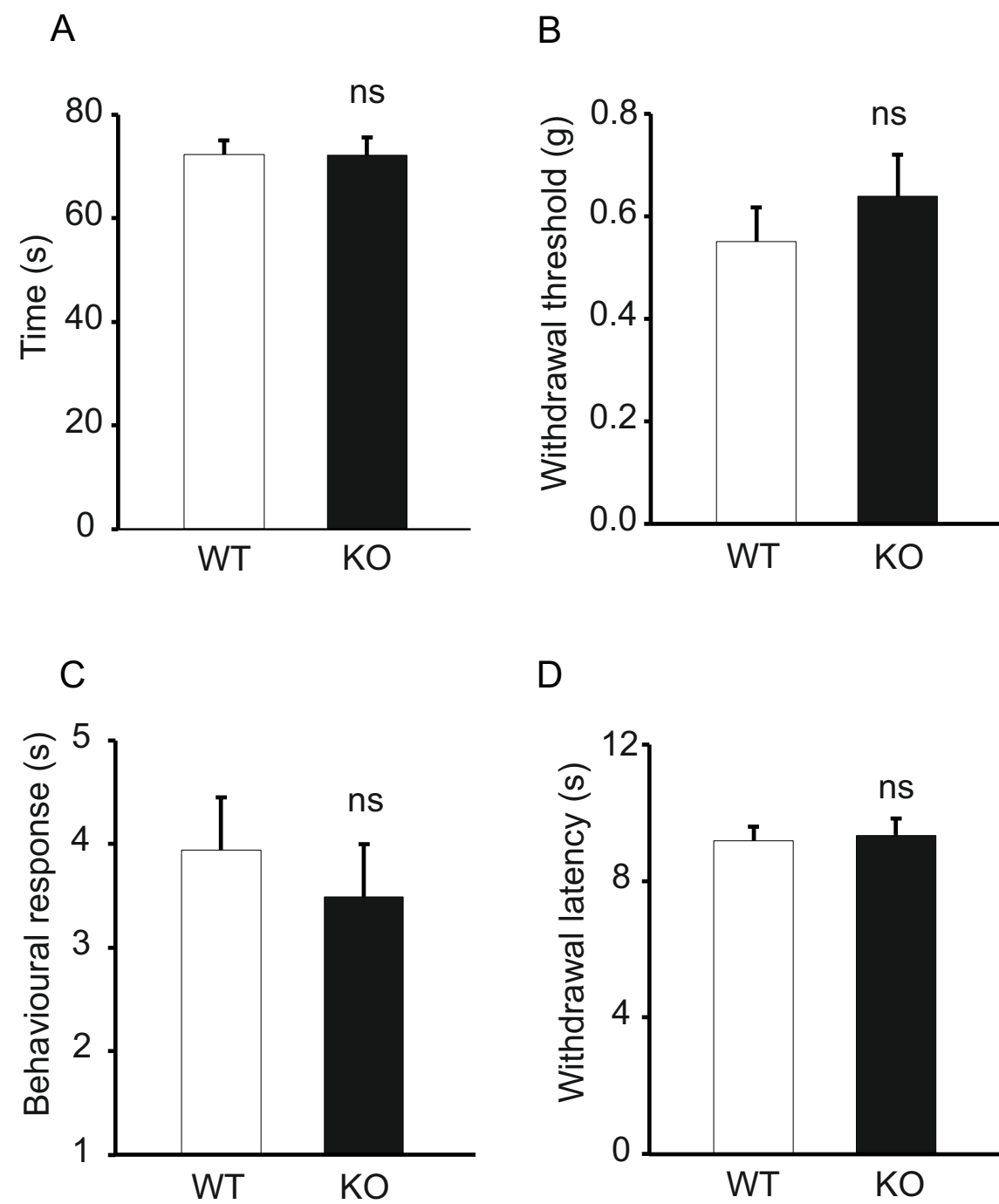

Figure S3

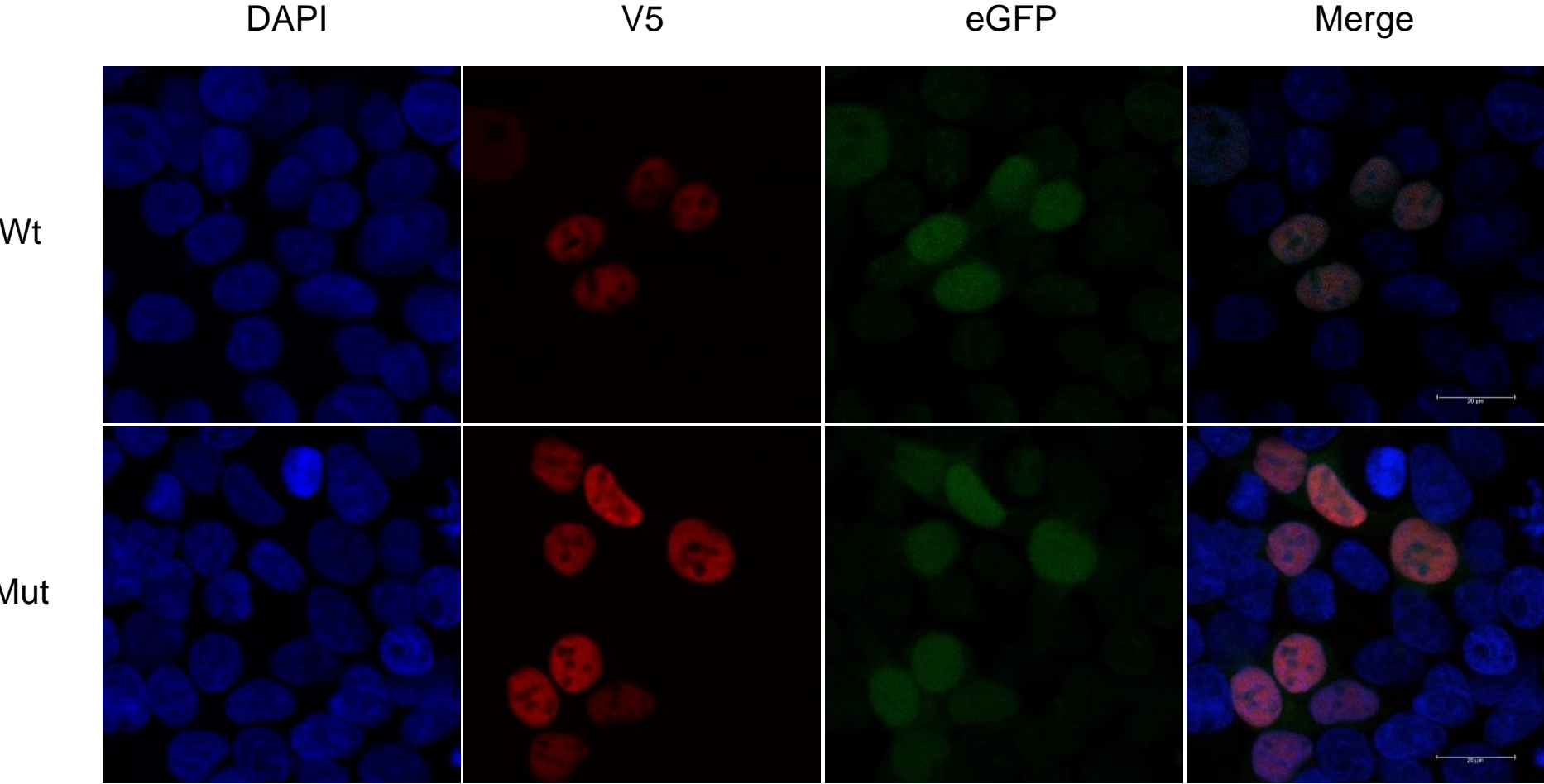

Figure S4

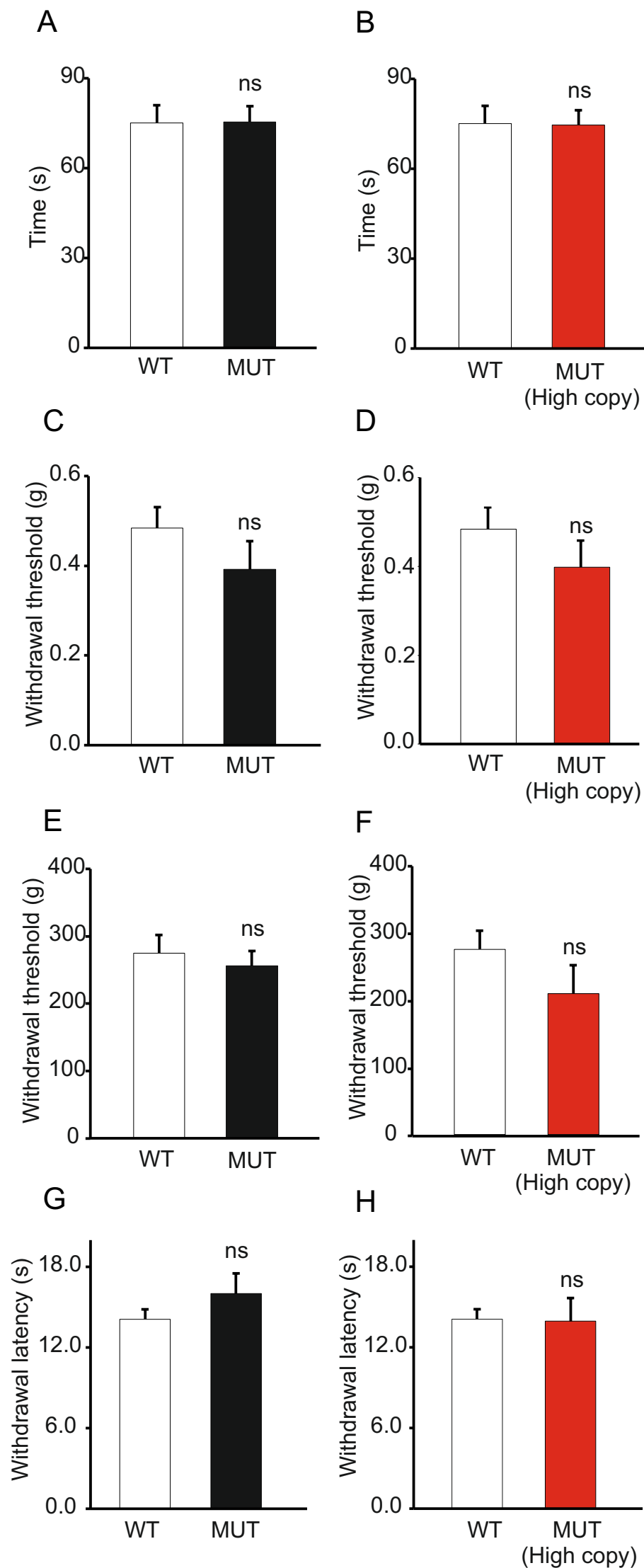

Figure S5

A

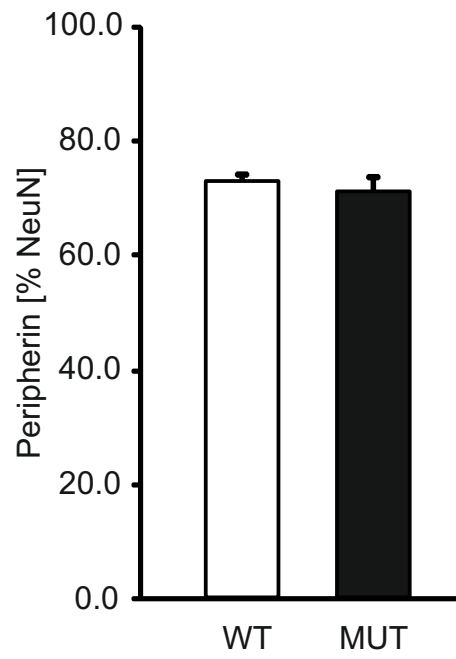

B

Anti-NeuN

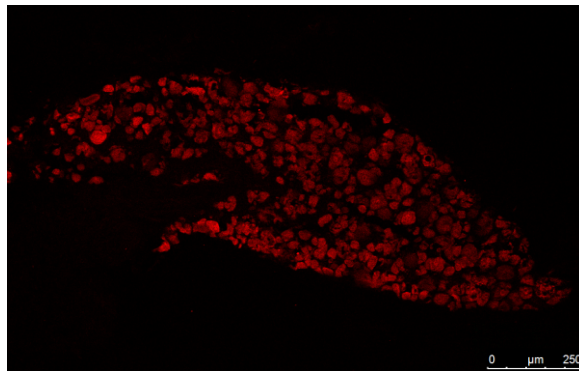

Anti-Peripherin

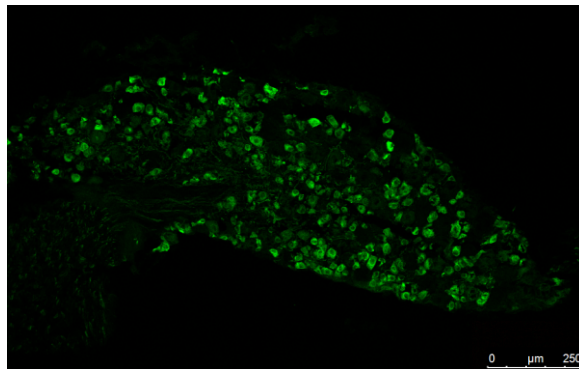

Merge

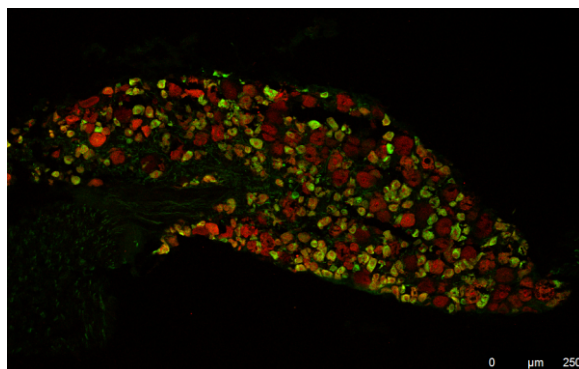

Figure S6

A

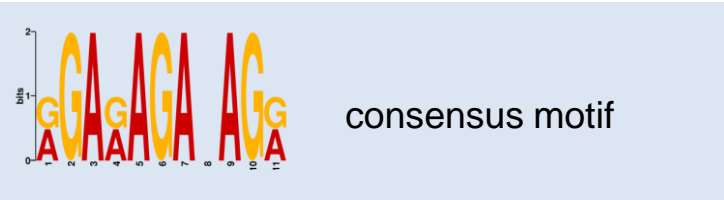

B

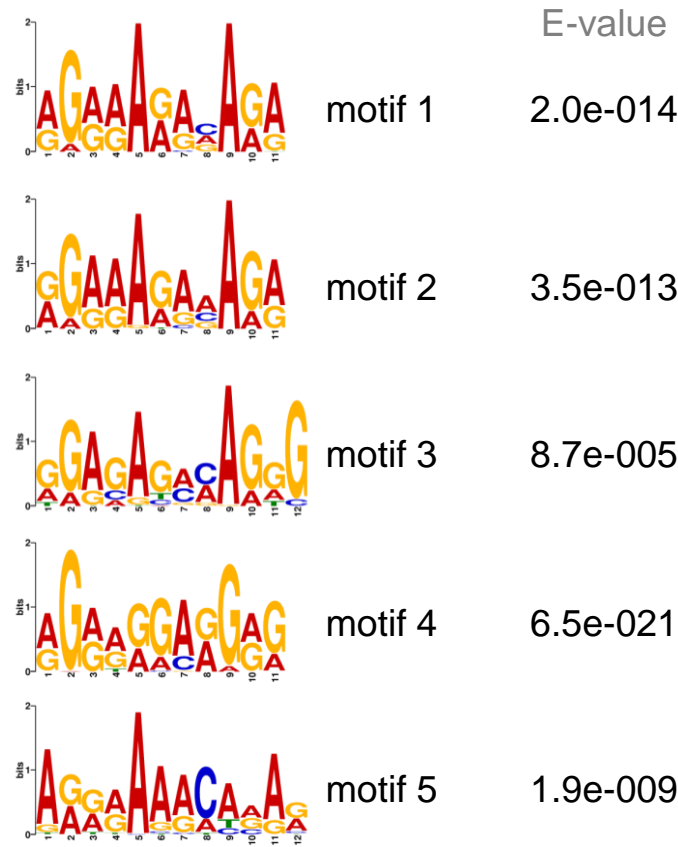

C

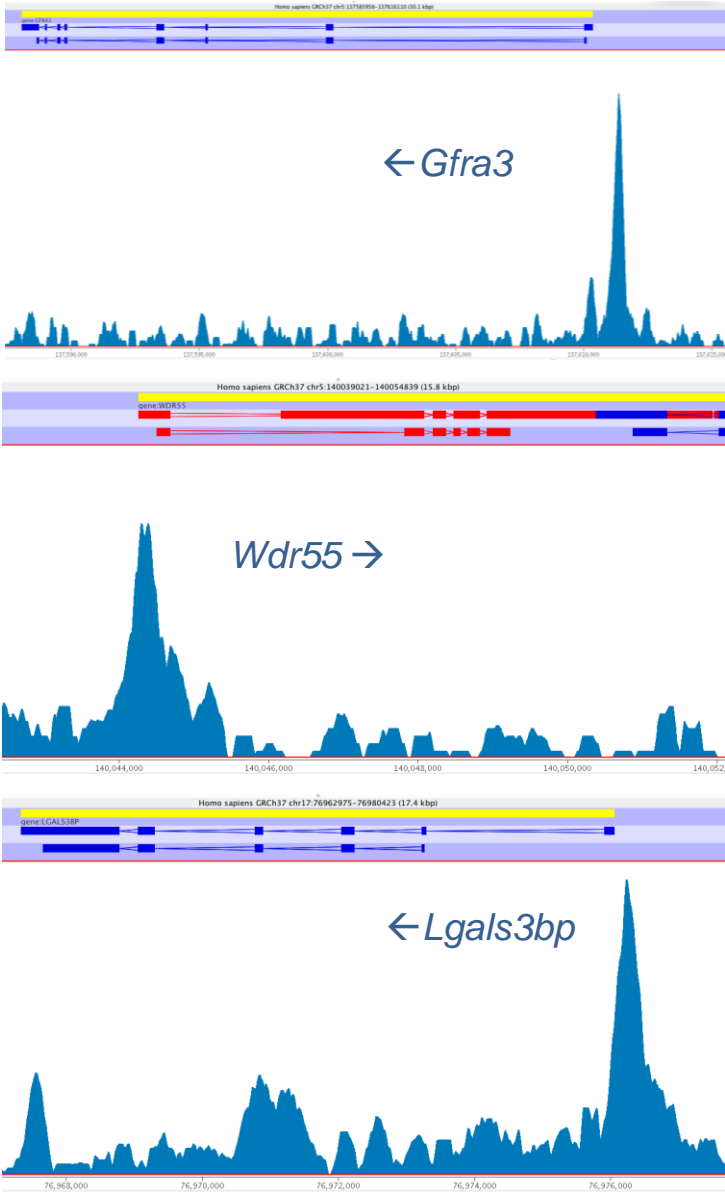

Supplement: Supplementary Figures [file Figures_S1-S6_awx326.pdf]
