# Supplementary material for: A novel human pain insensitivity disorder caused by a point mutation in ZFHX2
Source: Brain. 2017 Dec 14;141(2):365–76. doi: 10.1093/brain/awx326 (PMC5837393; doi:10.1093/brain/awx326)
Supplement: Supplemental Materials and Methods [file brain-2017-01205-File007_awx326.pdf]

Supplemental materials and methods

Real-time qPCR analysis in mouse tissues

One μg of total RNA derived from a range of tissues (Clontech) or extracted in-house from DRG, hypothalamus, thalamus and olfactory bulb tissues from C57BL/6 adult mice using the PureLink RNA Micro Kit (Invitrogen) was reverse transcribed according to standard protocols. Quantitative RT-PCR using technical triplicates was performed with the BioRad CXF Connect^{TM}. The PCR reaction mix consisted of first-strand cDNA template, primer pairs (Taqman probes for Zfhx2 (Mm01313868_m1) and Actb (Mm01205647_g1)) and, Master mix (Applied Biosystems). Zfhx2 expression was compared with that of *Actb* measured on the same sample in parallel on the same plate, giving a CT difference (ΔCT) for Actb minus the Zfhx2. Mean and standard error were performed on the ΔCT data and converted to relative expression levels (2ˆΔCT). Zfhx2 relative expression of individual tissues were compared to that of DRG. Data were analysed using Microsoft Excel.

Immunocytochemical analysis of dissociated DRG cultures

Following euthanization by inhalation of CO_2 and cervical dislocation, DRGs from adult wild-type C57BL/6 mice were dissected out and cultured as previously described, with the exception that poly-L-lysine and laminin-coated chamber slides (Thermo Scientific) were used instead of coated 35-mm dishes (Eijkelkamp *et al.*, 2013). After forty-eight hours in culture, the cells were fixed in 4% paraformaldehyde for 15 mins, washed in 1XPBS and then incubated for 10 mins in 1XPBS containing 0.25% Triton X-100 (PBST). Slides were then incubated in 3% goat serum in PBST at room temperature for 1 hr and then incubated with the following primary antibodies overnight at 4°C: rabbit polyclonal anti-Zfhx2 (1:100, from (Komine *et al.*, 2012)) and mouse monoclonal anti-peripherin (1:400, Sigma P5117). After three washes in PBST,

slides were incubated for 1 hr at room temperature in goat anti-rabbit Alexa Fluor 594 antibody (1:200, Thermo Fisher) and goat anti-mouse Alexa Fluor 488 antibody (1:200, Thermo Fisher). After 3 washes in PBST and 2 washes with PBS, the coverslips were mounted with VECTASHIELD Antifade Mounting Medium with DAPI (Vectorlabs). The cells were visualized using a spinning disk confocal microscope (Perkin Elmer).

Genotyping Zfhx2 knockout animals

Zfhx2 knockout animals (BRC No. 02262: B6.129S-Zfhx2<tm3Ymri>) were genotyped using the following PCR primers: WT allele product (913 bp) FWD (5’ CTA CCA TGG CTA CCC TTA ACT CA) and REV (5’ ACT GTG CTG GTG TCC GGT ACT TC); knockout allele product (620 bp) FWD (5’ GAG CTG GAC GGC GAC GTA AAC) and REV (5’ AAC TCC AGC AGG ACC ATG TG).

Expression constructs

The full-length human *ZFHX2* sequence was PCR amplified from a pool of adult human DRG, foetal brain and adult testis cDNA (Clontech) using KAPA HiFi Hotstart DNA Polymerase (Kapa Biosystems) and cloned into the *EcoR*I and *Xho*I restriction sites of pcDNA3 (Life Technologies) (primers available upon request). An N-terminal FLAG tag and a C-terminal V5 tag were inserted. Downstream of *ZFHX2*, an IRES-eGFP fragment was cloned into the *Xho*I and *Apa*I restriction sites. The R1913K mutation was inserted into the final clone using the QuikChange II XL site-directed mutagenesis kit (Agilent). Both constructs were verified by Sanger sequencing. The full-length *ZFHX2* sequenced has been deposited in GenBank with accession number KY781180.

Transient AD293 transfections and immunocytochemistry

FLAG-ZFHX2-V5-IRES-eGFP in pcDNA3 constructs (R1913-WT or K1913-MUT) were transfected into AD293 cells using Lipofectamine 2000 according to the manufacturer's conditions. Briefly, 2 μg of DNA was combined with 10 μl of Lipofectamine 2000 in Opti-Mem. Cells were transfected at 95% confluence and the following day were split onto Poly-L-Lysine coated glass coverslips. Forty-eight hours after transfection the cells were fixed with 4% PFA and V5-tagged ZFHX2 detected using a rabbit anti-V5 antibody (Sigma V8137, 1:500). The cells were visualized using a Leica SP8 confocal microscope.

Zfhx2 p.R1907K BAC transgenic mouse line

The Zfhx2 p.R1907K BAC transgenic line was generated by Cyagen Biosciences Inc. Briefly, BAC clone RP23-248M20 was modified by recombineering with incorporation of the *Zfhx2* point mutation (p.R1907K; codon AGG➔AAG). A male founder was produced following BAC purification and C57BL/6 pronuclear injection. Genotyping using primers at each end of the BAC sequence showed complete integration: 5' reaction, 336 bp (FWD: 5' AAG CGG ATG AAT GGC AGA AAT TCG, REV: 5' CCT ACA ATG TCA AGC TCG ACC GAT); 3' reaction, 450 bp (FWD: 5' GGG AAG GTT CAT ATG GCC CCT, REV: 5' CCT GGC CGT CGA CAT TTA GGT). The point mutation was confirmed following PCR amplification and Sanger sequencing: 593 bp (FWD: 5' CTC ATC AGT GAC CGG GAC A, REV: 5' CGC TTT CTG GAG ACG CTT TA).

Determining copy number of *Zfhx2* in genomic DNA of transgenic mice

Mouse ear biopsies were digested with lysis buffer for 2 hours at 55°C and 5 min at 95°C. The extracted genomic DNA was then further purified using the DNeasy Blood and Tissue Kit, as per the manufacturer's protocol (Qiagen). Real time PCR was carried out using the Universal SYBR Green Supermix protocol (Bio-Rad) and the following primers: *Zfhx2*, 229 bp (FWD: 5' CTA CCA TGG CTA CCC TTA ACT CA and REV: 3' GGC TCT CCA ATC TCC TTT GGT G) and *Dicer1*, 260 bp (FWD: 5' CTG GTG GCT TGA GGA CAA GAC and REV: 3' AGT GTA GCC TTA GCC ATT TGC). These assays were performed on the BioRad CXF Connect^{TM} real-time thermal cycler. Amplification reaction mixture (20 ul) for each gene target contained template genomic DNA (20 ng), final 1x concentration of Universal SYBR Green Supermix^{TM} (Bio-Rad), *Zfhx2* or the copy number reference *Dicer1* primer pairs. The cycling conditions used were 95°C for 2 min, followed by 40 cycles of 5 sec at 95°C and 65°C. Each experiment was run in triplicate with the copy number of *Zfhx2* and the reference *Dicer1* gene (2 genomic copies) measured on the same sample in parallel on the same plate, giving a CT difference (ΔCT) for *Dicer1* minus *Zfhx2*. The ΔCT data was converted to relative expression levels (2ˆΔCT) to give copy number per haploid genome and then multiplied by 2 to calculate diploid copy number. Whilst the copy number for *Dicer1* is constant at two in both the wild-type and transgenic animals, *Zfhx2* copy number is two only for the wild-type littermates.

Immunohistochemistry analysis of DRG

Fresh frozen unperfused DRG sections (from L4) were prepared as described previously (Zhao *et al.*, 2006). Three adult BAC transgenic animals with a 'high' genomic BAC copy number of 4 and three adult wild-type controls were used. DRGs were stained using anti-NeuN (Abcam,

Ab177487) and anti-peripherin (Sigma P5117). Sections were imaged using a Leica SP8 confocal microscope with positively stained neurons counted using ImageJ software.

Stable cell line creation

The human neuroblastoma cell line, SH-SY5Y (Public Health England) was cultured at 37°C/5% CO_2 in Ham's F12:DMEM (1:1) supplemented with 2 mM glutamine, 1% non essential amino acids and 10% foetal bovine serum (Life Technologies). The FLAG-ZFHX2 (K1913)-V5-IRES-eGFP mammalian expression plasmid was linearised with *Pvu*I and following gel purification (Qiagen) was transfected by Nucleofection (Lonza) into the cells and cultured for 3 days. Next, the cells were reseeded in complete media containing 500 □g/ml Geneticin (Life Technologies) and cultured for 16 days with the media replaced every 3 days. Single cells were selected by flow cytometry (BD FACSArray, SORP) using eGFP fluorescence into 96 well plates. Clones were cultured without Geneticin for 10 days, followed by media replacement with 500 □g/ml Geneticin. Two mutant clones were grown and validated by anti-V5 immunostaining and Sanger sequencing of genomic DNA and cDNA.

Motif Analysis

To construct a motif-binding model for ZFHX2, we used sequences from genes that were significantly deregulated in our microarray gene expression analysis of dorsal root ganglia samples from the BAC transgenic mice. The position frequency matrix of the ZFHX2 binding motif, termed AG motif, was determined using a mixture model by multiple EM (expectation maximization) for motif elicitation by MEME suite (http://meme-suite.org) (Bailey and Elkan, 1994). Promoter regions (1000 bp upstream and 50 bp downstream of the TSS) were extracted using Regulatory Sequence Analysis Tools (http://rsat.sb-roscoff.fr) and were searched for

motif occurrence in the differentially 119 expressed genes ($p<0.01$; Fold Change >1.2) from the combined analysis of microarray data (Table S3). The five best unique motifs (Fig. S6) were averaged into a consensus motif using STAMP (http://www.benoslab.pitt.edu/stamp/). Gene ontology search for gene families that contained the consensus motifs was performed by GOMo (http://meme-suite.org/tools/gomo).

Protein structure prediction

3D structure prediction for the ZFHX2 protein sequence was performed using PHYRE2 (http://www.sbg.bio.ic.ac.uk/~phyre2/html/page.cgi?id=index) (Kelley *et al.*, 2015). 3D models were further analysed by PyMol (http://pymol.sourceforge.net/) and UCSF Chimera (Pettersen *et al.*, 2004).

Chromatin immunoprecipitation (ChIP) assay

A stable transfected SH-SY5Y human neuroblastoma cell line expressing Zfhx2^{mut} at a high level was cultivated in 150mm petri dishes to 75-80% confluency. Cells were cross-linked with freshly prepared 37% formaldehyde to a final 1% concentration at RT on a slow rotating platform for 10 min and crosslinking was terminated by addition of ice cold glycine to a final concentration of 0.125 M for 5 min. Cells were harvested, washed in PBS and stored at -80°C. The chromatin from crosslinked cells was isolated as described (O'Sullivan *et al.*, 2002) and sheared to ~300 bp of average size fragments (six pulses of 10 min each at High setting on Bioruptor, Diagenode). Each sample had two technical replicas.

A single ChIP reaction used chromatin from 7.5x10^{7} cells and either anti-V5 (Abcam, ab15828, 0.7mg/ml) or anti-TBP for positive control (Abcam, ab28175, 1mg/ml) antibodies, incubated overnight at 4°C, then 2 h with M280 Sheep anti-Rabbit IgG Dynabeads (Invitrogen) at RT

typically using 5 µg of Ab and 25µl of Dynal beads per 1x10^6 of cells. Negative control ChIP reactions used rabbit IgG (Abcam).

Beads were washed 10 times in 450 µl volume of RIPA ChIP buffer (Dahl and Collas, 2008) and twice with 200 µl TE buffer with aid of the Precipitor (Abnova). DNA on washed beads was eluted and cross-link reversal was performed in one stage as described (Nelson *et al.*, 2006). DNA was purified with IPure kit (Diagenode) and Precipitor (Abnova). Final DNA samples were solubilised in 100 µl with concentration range between 7.5 to 14 ng/µl. The samples were used for library preparation and sequencing by Diagenode. The DNA sequencing was performed on an Illumina HiSeq 2500 (2 lanes, 50 bp, single end). Quality of sequencing reads was assessed using FastQC, alignment was performed using bwa v. 0.7.5a. Datasets were mapped to the human genome (GRCh37) and peak calling was performed using Seqmonk (http://www.bioinformatics.babraham.ac.uk/projects/seqmonk/). DNA sequence regions corresponding to DNA peaks were extracted using the UCSC genome browser (https://genome.ucsc.edu) and analysed by by MEME suite (http://meme-suite.org) (Bailey and Elkan, 1994). The raw data files have been submitted to ArrayExpress under accession number E-MTAB-5651.

References

Bailey TL, Elkan C. Fitting a mixture model by expectation maximization to discover motifs in biopolymers. Proc Int Conf Intell Syst Mol Biol 1994; 2: 28-36.

Dahl JA, Collas P. A rapid micro chromatin immunoprecipitation assay (microChIP). Nat Protoc 2008; 3(6): 1032-45.

Eijkelkamp N, Linley JE, Torres JM, Bee L, Dickenson AH, Gringhuis M, *et al.* A role for Piezo2 in EPAC1-dependent mechanical allodynia. Nature Communications 2013; 4.

Kelley LA, Mezulis S, Yates CM, Wass MN, Sternberg MJ. The Phyre2 web portal for protein modeling, prediction and analysis. Nat Protoc 2015; 10(6): 845-58.

Komine Y, Takao K, Miyakawa T, Yamamori T. Behavioral abnormalities observed in Zfhx2-deficient mice. PLoS One 2012; 7(12): e53114.

Nelson JD, Denisenko O, Bomsztyk K. Protocol for the fast chromatin immunoprecipitation (ChIP) method. Nat Protoc 2006; 1(1): 179-85.

O'Sullivan AC, Sullivan GJ, McStay B. UBF binding in vivo is not restricted to regulatory sequences within the vertebrate ribosomal DNA repeat. Mol Cell Biol 2002; 22(2): 657-68.

Pettersen EF, Goddard TD, Huang CC, Couch GS, Greenblatt DM, Meng EC, *et al.* UCSF Chimera--a visualization system for exploratory research and analysis. J Comput Chem 2004; 25(13): 1605-12.

Zhao J, Nassar MA, Gavazzi I, Wood JN. Tamoxifen-inducible NaV1.8-CreERT2 recombinase activity in nociceptive neurons of dorsal root ganglia. Genesis 2006; 44(8): 364-71.
