# Supplementary material for: A novel human pain insensitivity disorder caused by a point mutation in ZFHX2
Source: Brain. 2017 Dec 14;141(2):365–76. doi: 10.1093/brain/awx326 (PMC5837393; doi:10.1093/brain/awx326)
Supplement: Supplementary Table S1 [file brain-2017-01205-File009_awx326.pdf]

Table S1A: Examples of bone fractures in all six affected individuals

Individual (refer to Fig 1A)	Fracture	Approx age	Comments
**I-2**	Left foot metatarsal	53	Visited hospital 12 hrs later.
**II-2**	Right tibia	14	High pain tolerance with fracture.
**II-4 (proband)**	Right shoulder (scapula and clavicle)	46	Skied for 30 km for whole afternoon following fractures. Visited hospital following day.
**III-1**	Left wrist	12	Visited hospital following swelling, not due to high pain.
**III-2**	Right ankle	Between 8-22	Detected years later after calcification with magnetic resonance imaging.
**III-3**	Left elbow	7	Rode bicycle for 2 days following fracture without realizing break.

Table S1B: Sensory phenotype assessment data in four affected individuals

	Tender points (9+9 tender points and 5+5 control points)	Thermal detection pain threshold (^oC)	Cold pressor test (pain onset and tolerance, secs)	Mechanical detection threshold (von Frey filaments)
Proband (II-4)	0/18 and 0/10	-8.73 and 46.33	40 and 97	3.61#
II-2	3/18 and 1/10	-6.68 and 48.53	8** and 70	3.61#
I-2	0/18 and 0/10	-6.65 and 46.63	nd and 42	3.61#
III-2	1/18 and 0/10	-8.90 and 50.00*	70 and >155	3.61#

Table S1C: Mechanical pain threshold detection in the proband (II-4)

**Von Frey filaments evaluator size (target force, gms)**	**Sensation reported**
**4.56-4.93 (4-8 gms)**	Tactile sensation; no pain
**5.07 (10 gms)**	Pleasure (VAS20); no pain
**6.1-6.45 (100-180 gms)**	Pleasure (VAS30); no pain<br>Upon the 3rd repeat, the pleasant sensation was absent with only a tactile sensation perceived. Upon the 5th repeat, the tactile sensation was also lost. No pain throughout the tests.

Table S1D: Chemical pain evoked sensation (capsaicin test) in the proband (II-4)

Time	VAS	Main site reaction	Other site reactions
-10 min		(Basal tactile determination)<br>CW and VF filament (6.45 i.e. 180 gms): light touch and pleasant perception	
0		Injection	
15 sec		Pain onset	Head hot flushes
30 sec	80		
1 min	40		
1.30 min	0		Hand itching
2 min	0		Hand numbness
2 min 30 sec	0	Unpleasantness (VAS 30)	
3 min 30 sec	0	Small area of erythema at injection point	
4 min	0	Itching and pleasure at the site of injection	
5-6 min	0	CW + VF (2.83 i.e. 0.07 gms) only touch sensation stronger than those reported before injection<br>VF: in the periphery of the field she reports also a pleasurable sensation	Numb, coldness and prickle
