# Supplementary material for: A novel human pain insensitivity disorder caused by a point mutation in ZFHX2
Source: Brain. 2017 Dec 14;141(2):365–76. doi: 10.1093/brain/awx326 (PMC5837393; doi:10.1093/brain/awx326)
Supplement: Supplementary Data [file brain-2017-01205-File012_awx326.pdf]

1 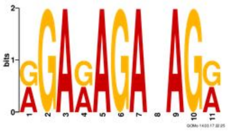 225

CC extracellular space
BP signal transduction
MF olfactory receptor activity
BP sensory perception of smell
BP cell adhesion

GO term	score	*p*-value	*q*-value	Spec-ty		GO name
GO:0007606	4.414e-05	1.030e-07	1.708e-05	25%	BP	sensory perception of chemical stimulus
GO:0007608	4.433e-05	1.030e-07	1.708e-05	~88%	BP	sensory perception of smell
GO:0007218	2.024e-02	1.498e-04	1.035e-02	100%	BP	neuropeptide signaling pathway
GO:0005125	8.439e-05	1.030e-07	1.708e-05	80%	MF	cytokine activity
GO:0003700	1.462e-03	1.030e-07	1.708e-05	~83%	MF	transcription factor activity
GO:0009897	2.181e-03	1.030e-07	1.708e-05	~53%	CC	external side of plasma membrane
GO:0005509	3.736e-03	8.240e-07	1.232e-04	100%	MF	calcium ion binding
GO:0060442	6.050e-03	2.781e-06	3.522e-04	~89%	BP	branching involved in prostate gland morphogenesis
GO:0060512	8.954e-03	1.184e-05	1.227e-03	~55%	BP	prostate gland morphogenesis
GO:0060740	9.274e-03	1.298e-05	1.315e-03	~67%	BP	prostate gland epithelium morphogenesis
GO:0048486	9.533e-03	1.401e-05	1.404e-03	32%	BP	parasympathetic nervous system development
GO:0021936	1.088e-02	2.173e-05	1.962e-03	~62%	BP	regulation of granule cell precursor proliferation
GO:0021940	1.088e-02	2.173e-05	1.962e-03	~86%	BP	positive regulation of granule cell precursor proliferation
GO:0048663	1.403e-02	4.892e-05	4.131e-03	~19%	BP	neuron fate commitment
GO:0022843	1.443e-02	5.438e-05	4.549e-03	~52%	MF	voltage-gated cation channel activity
GO:0051480	1.537e-02	6.592e-05	5.319e-03	~67%	BP	cytosolic calcium ion homeostasis
GO:0032330	1.548e-02	6.788e-05	5.429e-03	80%	BP	regulation of chondrocyte differentiation
GO:0005539	1.666e-02	8.683e-05	6.825e-03	~45%	MF	glycosaminoglycan binding
GO:0008009	1.743e-02	9.836e-05	7.521e-03	100%	MF	chemokine activity
GO:0006874	1.987e-02	1.418e-04	9.948e-03	~44%	BP	cellular calcium ion homeostasis
GO:0007601	2.118e-02	1.719e-04	1.161e-02	~47%	BP	visual perception
GO:0050953	2.175e-02	1.849e-04	1.240e-02	~35%	BP	sensory perception of light stimulus
GO:0021569	2.213e-02	1.944e-04	1.294e-02	80%	BP	rhombomere 3 development
GO:0007435	2.267e-02	2.101e-04	1.359e-02	50%	BP	salivary gland morphogenesis
GO:0021937	2.289e-02	2.165e-04	1.377e-02	~94%	BP	cerebellar Purkinje cell-granule cell precursor cell signaling involved in regulation of granule cell precursor cell proliferation
GO:0021783	2.317e-02	2.240e-04	1.409e-02	40%	BP	preganglionic parasympathetic nervous system development
GO:0048483	2.338e-02	2.317e-04	1.447e-02	~23%	BP	autonomic nervous system development
GO:0060235	2.408e-02	2.542e-04	1.577e-02	100%	BP	lens induction in camera-type eye
GO:0006875	2.568e-02	3.130e-04	1.846e-02	~30%	BP	cellular metal ion homeostasis
GO:0021516	2.570e-02	3.137e-04	1.846e-02	60%	BP	dorsal spinal cord development
GO:0021527	2.605e-02	3.261e-04	1.901e-02	85%	BP	spinal cord association neuron differentiation
GO:0007204	2.608e-02	3.273e-04	1.901e-02	76%	BP	elevation of cytosolic calcium ion concentration
GO:0004981	2.632e-02	3.362e-04	1.940e-02	100%	MF	muscarinic acetylcholine receptor activity
GO:0002052	2.683e-02	3.569e-04	2.034e-02	100%	BP	positive regulation of neuroblast proliferation
GO:0008528	2.758e-02	3.870e-04	2.178e-02	~14%	MF	peptide receptor activity, G-protein coupled
GO:0002089	2.952e-02	4.709e-04	2.571e-02	~88%	BP	lens morphogenesis in camera-type eye
GO:0004866	3.018e-02	5.041e-04	2.652e-02	40%	MF	endopeptidase inhibitor activity
GO:0031420	3.081e-02	5.343e-04	2.766e-02	~63%	MF	alkali metal ion binding
GO:0030023	3.089e-02	5.400e-04	2.766e-02	100%	MF	extracellular matrix constituent conferring elasticity
GO:0045211	3.100e-02	5.456e-04	2.777e-02	100%	CC	postsynaptic membrane
GO:0004867	3.361e-02	6.966e-04	3.415e-02	100%	MF	serine-type endopeptidase inhibitor activity
GO:0002088	3.498e-02	7.870e-04	3.730e-02	~63%	BP	lens development in camera-type eye
GO:0001992	3.502e-02	7.896e-04	3.730e-02	~86%	BP	regulation of systemic arterial blood pressure by vasopressin
GO:0010810	3.530e-02	8.079e-04	3.759e-02	~38%	BP	regulation of cell-substrate adhesion
GO:0048505	3.558e-02	8.263e-04	3.768e-02	70%	BP	regulation of timing of cell differentiation
GO:0045785	3.561e-02	8.285e-04	3.768e-02	~39%	BP	positive regulation of cell adhesion
GO:0030901	3.564e-02	8.307e-04	3.768e-02	~91%	BP	midbrain development
GO:0002053	3.640e-02	8.878e-04	3.988e-02	90%	BP	positive regulation of mesenchymal cell proliferation
GO:0021515	3.701e-02	9.337e-04	4.113e-02	~31%	BP	cell differentiation in spinal cord
GO:0021561	3.775e-02	9.920e-04	4.232e-02	~76%	BP	facial nerve development
GO:0021604	3.775e-02	9.920e-04	4.232e-02	50%	BP	cranial nerve structural organization
GO:0021610	3.775e-02	9.920e-04	4.232e-02	~89%	BP	facial nerve morphogenesis
GO:0021612	3.775e-02	9.920e-04	4.232e-02	100%	BP	facial nerve structural organization
GO:0032982	3.790e-02	1.004e-03	4.256e-02	~94%	CC	myosin filament
GO:0001642	3.823e-02	1.032e-03	4.307e-02	100%	MF	group III metabotropic glutamate receptor activity
GO:0005244	3.831e-02	1.039e-03	4.307e-02	~39%	MF	voltage-gated ion channel activity
GO:0022832	3.831e-02	1.039e-03	4.307e-02	~23%	MF	voltage-gated channel activity
GO:0010464	3.852e-02	1.058e-03	4.365e-02	~42%	BP	regulation of mesenchymal cell proliferation
GO:0045346	3.917e-02	1.121e-03	4.604e-02	~78%	BP	regulation of MHC class II biosynthetic process
GO:0021570	3.958e-02	1.153e-03	4.716e-02	80%	BP	rhombomere 4 development

GOMo version: 4.11.3 (Release date: Sat Mar 11 20:16:08 2017 -0800)
Significance Threshold: 0.05
References: Mikael Bodén and Timothy L. Bailey, (2008) "Associating transcription factor binding site motifs with target Go terms and target genes", *Nucleic Acids Research*, **36**, 4108-4117.
Fabian A. Buske, Mikael Bodén, Denis C. Bauer and Timothy L. Bailey, (2010) "Assigning roles to DNA regulatory motifs using comparative genomics", *Bioinformatics*, **26**, 860-866.
