# Supplementary material for: A novel human pain insensitivity disorder caused by a point mutation in ZFHX2
Source: Brain. 2017 Dec 14;141(2):365–76. doi: 10.1093/brain/awx326 (PMC5837393; doi:10.1093/brain/awx326)
Supplement: Supplementary Data [file brain-2017-01205-File015_awx326.pdf]

Fig. S1. 3D structure motifs in ZFHX2 protein

(A) Amino acid sequence of ZFHX2 protein with seventeen Zn-finger motifs highlighted in blue, Prdm9-like domain highlighted in green, and three homeodomains highlighted in orange (amino acid numbering is shown in B). The conserved Arg 1913 residue is shown in magenta and indicated by an asterisk. (C) The Prdm9-like domain of ZFHX2 modelled on the SET domain of PRDM9 (99.9% confidence; PDB: 4ijd), Blue sphere indicates Zn atom of the C-terminal Zn-finger which is a part of the domain. (D) 3D model of the ZFHX2's second homeodomain (helix-turn-helix DNA-binding domain) (aa 1857-1919) based on Homeobox protein BarH-like 1 (93% coverage, 99.9% confidence; PDB: 2dmtA). The mutated Arg1913 position is shown in magenta.

Fig. S2. Further behaviour tests in *Zfhx2* global knockout mice

(A) Rotarod test showing no significant differences in motor co-ordination between WT (n=14) and KO (n=16) mice, p=0.97

(B) Von Frey test showing no significant differences in innocuous mechanical sensitivity between WT (n=14) and KO (n=16) mice, p=0.41.

(C) Acetone evaporation test showing no significant differences in mild cooling between WT (n=14) and KO (n=16) mice, p=0.53.

(D) Hargreaves' test showing no significant differences in withdrawal latencies between WT (n=18) and KO (n=18) mice, p=0.81. All data analysed by t-test, results are presented as mean±s.e.m.; ns p>0.05.

Fig. S3. Nuclear localization of wild-type and mutant ZFHX2 in AD293 cells

Wild-type (R1913, upper panel) and mutant (K1913, lower panel) FLAG-ZFHX2-V5-IRES-eGFP expression constructs were transiently transfected into AD293 cells. Both the wild-type and mutant ZFHX2 proteins (anti-V5 in red) localize to the nucleus (DAPI in blue) in positively transfected cells (eGFP fluorescence in green). Scale bar 20 μm.

Fig. S4. Further behaviour tests in *Zfhx2* p.R1907K BAC transgenic mice

(A, B) Rotarod test showing no significant differences in motor co-ordination between WT (n=16) and (A) mutant (n=16, genomic BAC copy number 1-5, p=0.964) and (B) high-copy mutant (n=5, genomic BAC copy number 4-5, p=0.967) mice.

(C, D) Von Frey test showing no significant differences in innocuous touch sensation between WT (n=16) and (C) mutant (n=16, genomic BAC copy number 1-5, p=0.251) and (D) high-copy mutant (n=5, genomic BAC copy number 4-5, p=0.35) mice.

(E, F) Randall Selitto test measuring withdrawal thresholds in the tail to noxious mechanical stimuli shows no significant differences between WT (n=16) and (E) mutant (n=16, genomic BAC copy number 1-5, p=0.61) and (F) high-copy mutant (n=5, genomic BAC copy number 4-5, p=0.242) mice.

(G, H) Cold plantar assay measuring withdrawal thresholds in the paw to noxious cold shows no significant differences between WT (n=16) and (G) mutant (n=16, genomic BAC copy number 1-5, p=0.268) and (H) high-copy mutant

(n=5, genomic BAC copy number 4-5, p=0.483) mice. All data analysed by t-test, results are presented as mean±s.e.m.; ns p>0.05.

Fig. S5. Immunohistochemical analysis of DRG from WT and *Zfhx2* p.R1907K BAC transgenic mice

(A) Counts of cells stained for the small diameter unmyelinated neuronal marker peripherin and the general neuronal marker NeuN showed no significant differences in the proportion of peripherin-positive DRG neurons in wild-type (n=3) and mutant BAC transgenic mice (n=3, genomic copy number of 4, p=0.5). Data analysed by t-test, results are presented as mean±s.e.m.; ns p>0.05.

(B) Example staining of a mouse DRG section used for cell counts with NeuN in red and peripherin in green. Scale bar 250 μm.

Fig. S6. Motif and ChIP-seq analyses

The consensus motif (A) has been constructed using 5 common motifs (B) from the deregulated genes in the DRG microarray screen. Motif 1 was obtained from ChIP-seq peak areas enriched in ZFHX2 binding, examples in (C).

Table S1. Sensory phenotype assessment data

(A) Examples of bone fractures in all six affected individuals.

(B) Sensory phenotype assessment data in four affected individuals.

In the proband (Fig. 1A), none of the tender or control points were positive for the deep pressure pain stimulation. In all individuals tested the heat pain thresholds were greater than 46°C, with one individual (III-2) having a very high pain threshold (no pain at 50°C). Cold pain thresholds: subjects were hyposensitive in relation to cold pain as shown by the low temperature needed to evoke pain. All individuals showed normal mechanical detection thresholds of innocuous stimuli.

Individuals as denoted in Fig. 1A; * at 50°C still no pain; ** hyperalgesia at the threshold; nd, value not determined; #, value in grams=0.4 gms.

(C) Mechanical pain threshold detection in the proband (II-4)

(D) Chemical pain evoked sensation (capsaicin test) in the proband (II-4)

Basal: Preliminary determination of the sensorial characteristics of the skin reported a light touch perception for the cotton wool (CW) while with the von Frey (VF) filament (6.45 i.e. 180 gms) she reported pleasant perception (VAS30).

Capsaicin injection: During capsaicin injection, lasting a few secs, the proband reported an immediate and very intense pain (VAS80) that suddenly disappeared as reported in detail in the table.

Table S2. Differentially expressed DRG genes

Table summarizes the genes differentially expressed in lumbar DRG (L1-6) between *Zfhx2* p.R1907K BAC transgenic mice (n=5, genomic BAC copy number of 4) and wild-type controls (n=7). Genes with a fold change of at least 20% between genotypes and with an Anova p value cut-off of ≤ 0.01 are shown. In bold are genes located in the BAC; genes highlighted in yellow are upregulated or downregulated at least 1.7 fold.

Table S3. Genes used for motif analysis

List of genes used in the promoter region analysis by MEME suite (http://meme-suite.org). Genes containing ChIP-seq peaks are annotated in column D.

Table S4. Gene ontology enrichment for genes containing the AG-rich consensus motif

This table is related to Fig. 5. Profiler was used to find the enriched biological process terms and molecular function for genes containing the AG-rich consensus motif. BP - biological process, CC - cellular component, MF - molecular function.
